# Super‐Soft DNA/Dopamine‐Grafted‐Dextran Hydrogel as Dynamic Wire for Electric Circuits Switched by a Microbial Metabolism Process

**DOI:** 10.1002/advs.202000684

**Published:** 2020-05-25

**Authors:** Jinpeng Han, Yuchen Cui, Xinpeng Han, Chenyu Liang, Wenguang Liu, Dan Luo, Dayong Yang

**Affiliations:** ^1^ Frontier Science Center for Synthetic Biology Key Laboratory of Systems Bioengineering (MOE) School of Chemical Engineering and Technology Tianjin University Tianjin 300350 P. R. China; ^2^ Tianjin Key Laboratory of Composite and Functional Materials School of Materials Science and Engineering Tianjin University Tianjin 300350 P. R. China; ^3^ Department of Biological & Environmental Engineering Cornell University Ithaca NY 14853 USA

**Keywords:** DNA hydrogels, electric circuits, microbial metabolism, synthetic biology, volume responsiveness

## Abstract

Engineering dynamic systems or materials to respond to biological process is one of the major tasks in synthetic biology and will enable wide promising applications, such as robotics and smart medicine. Herein, a super‐soft and dynamic DNA/dopamine‐grafted‐dextran hydrogel, which shows super‐fast volume‐responsiveness with high sensitivity upon solvents with different polarities and enables creation of electric circuits in response to microbial metabolism is reported. Synergic permanent and dynamic double networks are integrated in this hydrogel. A serials of dynamic hydrogel‐based electric circuits are fabricated: 1) triggered by using water as switch, 2) triggered by using water and petroleum ether as switch pair, 3) a self‐healing electric circuit; 4) remarkably, a microbial metabolism process which produces ethanol triggering electric circuit is achieved successfully. It is envisioned that the work provides a new strategy for the construction of dynamic materials, particularly DNA‐based biomaterials; and the electric circuits will be highly promising in applications, such as soft robotics and intelligent systems.

## Graphical abstract

A super‐soft and dynamic DNA‐based hybrid hydrogel is reported, which shows super‐fast volume‐responsiveness with high sensitivity upon solvents with different polarities and enables creation of electric circuits in response to microbial metabolism.

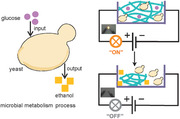

## Introduction

Dynamic hydrogels stimulated by specific stimuli or biological process have emerged as potential candidates for a wide range of applications, such as soft robots,^[^ ^1^, ^2^ ^]^ next‐generation bioelectronics interfaces,^[^ ^3^, ^4^ ^]^ switchable catalysis,^[^ ^5^ ^]^ and targeted gene therapy.^[^ ^6^ ^]^ Many polymer materials have been used for the construction of dynamic hydrogels with reversible volume and shape changes, due to the presence of entropic elasticity of polymer chains.^[^ ^7^, ^8^ ^]^ The general strategy for designing dynamic hydrogels is constructing synergic permanent and temporary double networks. The permanent networks created by covalent bonds maintained the structural integrity; meanwhile the temporary networks created by dynamic interactions were responsible for the responsiveness upon stimuli.^[^ ^7^, ^9^, ^10^ ^]^

DNA composed of four deoxyribonucleotide monomers was regarded as a block copolymer and showed great potential for the construction of dynamic soft DNA materials, especially for dynamic soft DNA hydrogels (lower than 100 Pa of elastic modulus).^[^ ^11^ ^]^ The ultrahigh molecular weights of DNA enabled the entropic elasticity of DNA copolymers.^[^ ^12^ ^]^ Therefore, DNA was a competitive alternative for the construction of dynamic DNA hydrogels in response to specific stimuli or biological process.^[^ ^13^, ^14^, ^15^ ^]^ For example, Luo and colleagues synthesized a dynamic DNA hydrogel, which exhibited solid/liquid transition in response to water.^[^ ^16^ ^]^ Liu and colleagues prepared an enzyme‐responsive DNA hydrogel possessing double networks.^[^ ^17^ ^]^ Fan and colleagues developed a DNA hydrogel with volume changes in response to specific DNA sequence.^[^ ^18^ ^]^ Willner and colleagues reported a pH‐responsive DNA hybrid hydrogel with multiple shape‐memory properties.^[^ ^19^ ^]^ Besides, the unique attributes of DNA molecules, such as the intrinsic biological functions, molecular recognition, sequence programmability, and biocompatibility^[^ ^20^, ^21^, ^22^ ^]^ endowed DNA hydrogels with rationally designed molecular structures and diversified biofunctions. For instance, Tan and colleagues prepared redox‐responsive DNA nanogels for targeted gene therapy.^[^ ^6^ ^]^ Schulman and colleagues fabricated shape‐changing DNA hydrogels for programmable soft devices.^[^ ^1^ ^]^

Recently, we have demonstrated that the physical interactions of long DNA chains can result in the formation of super‐soft DNA hydrogels. For example, we prepared a super‐soft and super‐elastic magnetic DNA hydrogel‐based robot (DNA robot) via utilizing DNA chain entanglement and hybridization for the construction of combinational dynamic and permanent crosslinking networks.^[^ ^2^ ^]^ This DNA robot showed shape‐adaptive properties and thus enabled the magnetically driven navigational locomotion for cell delivery in unstructured and confined space. We further constructed a DNA network via double rolling circle amplification, and through the intertwining and self‐assembly of two strands of ultralong DNA chains.^[^ ^22^ ^]^ This special structural design endowed DNA hydrogel with the desired functions and properties to fulfill the stem cell fishing and controlled release. In general, to construct dynamic DNA hydrogels in response to specific stimuli, designing synergic permanent and temporary double networks was proposed, in which the permanent networks maintained the structural integrity, meanwhile the temporary networks were responsible for the responsiveness. Inspired by the adhesive proteins in mussel byssus,^[^ ^23^ ^]^ dopamine (DOPA)‐functionalized polymer has been served as “molecular glue” to prepare dynamic hydrogels.^[^ ^24^, ^25^ ^]^ DOPA‐mediated hydrogen bonds and the subsequent covalent crosslinking were responsible for the formation of temporary and permanent networks, respectively.^[^ ^26^ ^]^ In particular, it has been demonstrated that the strong interactions between DOPA and DNA had significant effect on the conformation of DNA nanostructures, showing great potential for constructing DOPA‐based DNA networks.^[^ ^27^ ^]^

Herein, we report a super‐soft and dynamic nanofiber‐assembled DNA/dopamine‐grafted‐dextran hydrogel composed of natural long DNA chains and dopamine‐grafted‐dextran (DEX‐g‐DOPA), which showed super‐fast volume‐responsiveness with high sensitivity upon solvents with different polarities. Synergic permanent and dynamic double networks were integrated in this hydrogel. DOPA–DOPA covalent interactions provided permanent crosslinking for hydrogel framework; meanwhile hydrophobic interactions, hydrogen bonds, and π–π stacking between DNA and DEX‐g‐DOPA formed dynamic interactions. By replacing solvents, the volume of hydrogel changed within a few seconds because the hydrogel was super‐soft. Although the change of solvent polarity was only 0.4, the volume change of hydrogel was visually distinguished, demonstrating the high sensitivity of the responsiveness. Regarding the previously reported solvent‐responsive hydrogels (Table S1, Supporting Information), the modulus of the hydrogels was usually higher than 1000 Pa, which usually needed a relatively long time to achieve the volume changes, because the hydrogels with high modulus and crosslinking density were not favor to the rapid exchange of solvents.^[^ ^28^ ^]^ By using this unique DNA hydrogel as dynamic connecting wires, a serials of dynamic hydrogel‐based electric circuits were fabricated: 1) triggered by using water as switch, 2) triggered by using water and petroleum ether as switch pair, 3) a self‐healing electric circuit; 4) remarkably, a microbial metabolism process which produced ethanol triggering electric circuit was achieved successfully. To the best of our knowledge, this is the first example of the electric circuits switched by a dynamic microbial metabolism process without human interventions.

## Results and Discussion

**Scheme 1:**
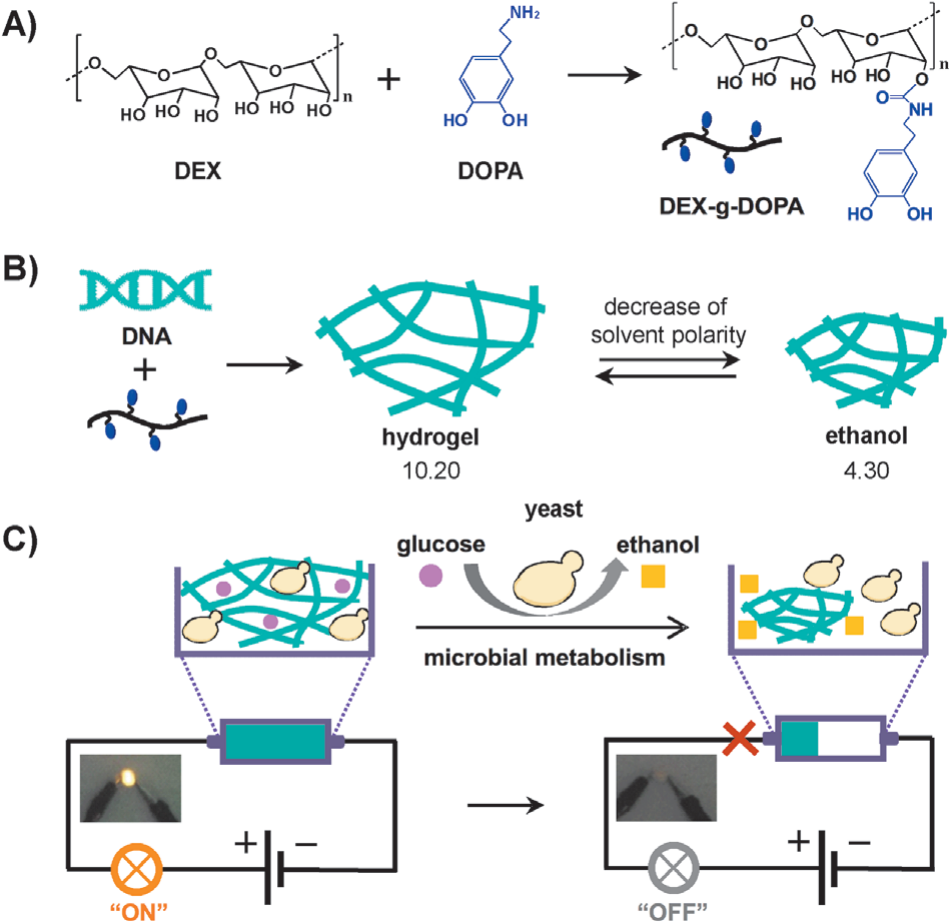
Molecular design and synthesis route of super‐soft and dynamic DNA/DEX‐g‐DOPA hydrogel. A) The synthesis route of DEX‐g‐DOPA. B) Preparation of the nanofiber‐assembled hydrogel with volumeric responsiveness upon solvent polarity. C) Electric circuit switched by a microbial metabolism process which produced ethanol using DNA/DEX‐g‐DOPA hydrogel as dynamic wires.

**Scheme** **1** describes the overall molecular design and synthesis route of DNA/DEX‐g‐DOPA hydrogel. First, DEX‐g‐DOPA was synthesized via the grafting DOPA onto the backbone of dextran (DEX, 6000 Da) (Scheme 1A). The hydroxyl groups of DEX reacted with 1,1’‐carbonyldiimidazole (CDI) to form imidazolyl carbamates, which were further coupled with the primary amine groups of DOPA, thus yielding DEX‐g‐DOPA. ^1^H Nuclear magnetic resonance (^1^H NMR), ultraviolet‐visible light (UV–vis), and Fourier transform infrared (FT‐IR) spectrum confirmed the structure of DEX‐g‐DOPA (Figure S1, Supporting Information). From ^1^H NMR results, the grafting rate of DEX‐g‐DOPA was ≈11.1%. Then natural salmon sperm DNA was mixed with DEX‐g‐DOPA, resulting in the formation of nanofiber‐assembled DNA/DEX‐g‐DOPA hydrogel through multiple interactions (Scheme 1B). The molecular weight of the used DNA was ≈1.2 × 10^7^ Da (20 000 bp), which was calculated from the agarose gel electrophoresis results (Figure S2, Supporting Information). By regulating the solvent polarity, the volume of the hydrogel could be precisely controlled. Moreover, the volume of the hydrogel showed a positive linear correlation with the solvent polarity. Owing to this special property, we noticed that a variety of microorganisms were able to produce solvents with different polarities, such as ethanol, acetic acid, butanol, and acetone. As a demonstration of application, a microbial metabolism process which produced ethanol triggering electric circuit was achieved using DNA/DEX‐g‐DOPA hydrogel as dynamic wires (Scheme 1C). Notably, though the maximum production of ethanol during microbial metabolism process was 10% at most and thus the change of solvent polarity was only 0.59, our prepared hydrogel with super‐fast volume‐responsiveness with high sensitivity upon the change of solvent polarity was utilized to dynamically switch the electric circuit.

**Figure 1:**
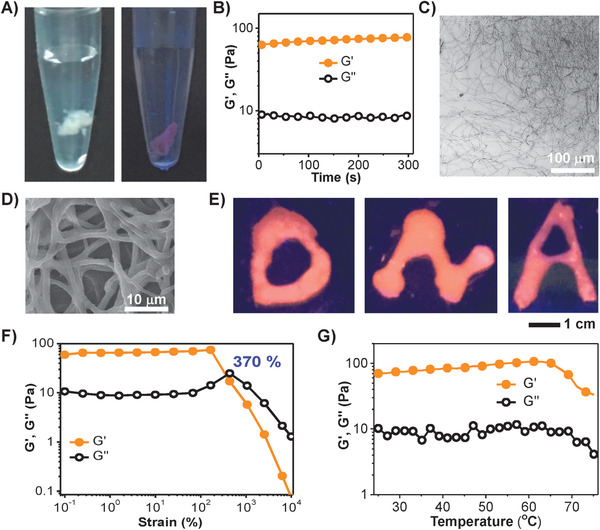
Formation of nanofiber‐assembled DNA/DEX‐g‐DOPA hydrogel. The content of DNA and of DEX‐g‐DOPA in the hydrogel was 67 w/v% and 33 w/v%, respectively. A) Digital photos of DNA/DEX‐g‐DOPA hydrogel stained with GelRed, a DNA‐specific dye. B) Storage (G′) and loss (G″) modulus of the hydrogel as a function of time, indicating the gel property of hydrgel. C) Fluorescence image of the nanofiber‐assembled hydrogel. D) SEM image of the hydrogel. E) D‐, N‐, and A‐shaped hydrogels stained with GelRed were written using a syringe, suggesting that the hydrogel was injectable. F) G′ and G″ of the hydrogel as a function of strain, demonstrating that gel‐to‐liquid transition occurred when the strain was 370%. G) G′ and G″ of the hydrogel as a function of temperature, demonstrating the stability of hydrogel in a wide range of temperatures.

When DNA and DEX‐g‐DOPA were mixed together at 90 °C and incubated at room temperature for 5 days, an opaque DNA/DEX‐g‐DOPA hydrogel formed spontaneously in test tube (**Figure** **1**A). The hydrogel was stained with a DNA specific fluorescent dye, GelRed and showed red fluorescence under ultraviolet light, suggesting that the entire hydrogel was composed of DNA. Rheology results further confirmed the formation of hydrogel, as the storage modulus (G′) was constantly higher than loss modulus (G″) (Figure 1B). The G′ value was ≈59 Pa, showing the super‐soft mechanical strength. It was notable that the hydrogel was composed of entangled nanofibers (Figure 1C). Under fluorescence microscope, the hydrogel showed apparent fibrous structures, wherein DNA components of the hydrogel was specifically stained green using SYBR Green I, demonstrating the formation of DNA‐based nanofibers (Figure S3, Supporting Information). Scanning electron microscope (SEM) image gave more details that nanofibers were relatively uniform and the diameter was ≈800 nm (Figure 1D). Considering the hydrogel which was composed of entangled nanofibers, the hydrogel was injectable through the motions and rearrangements of nanofibers. Therefore, we used a syringe containing hydrogel to write any arbitrary shapes, such as the letters of D, N, and A with red fluorescence (Figure 1E). Besides, due to the relatively low cost of natural DNA, the hydrogel showed high potential in scale‐up industrial production.

Under strain sweep mode, when the strain was less than 370%, G′ value was constantly higher than G″ value, showing the gel property. When the strain was larger than 370%, the G″ value turned to be higher than G′ value, showing the liquid property (Figure 1F). G″ value increased slightly when the strain changed from 100% to 370%, because the sliding of DNA/DEX‐g‐DOPA nanofibers needed to dissipate energy.^[^ ^29^ ^]^ Under temperature sweep mode, the hydrogel was relatively stable to maintain the gel properties over entire temperature range, from 25 to 75 °C (Figure 1G). The influence factors for the formation of hydrogel were discussed in detail (Discussion S1, Supporting Information). Among DEX, oxidized DEX (aldehyde groups grafted DEX) and DEX‐g‐DOPA, only DEX‐g‐DOPA was able to interact with DNA to form the hydrogel (Figure S4, Supporting Information). Moreover, the appropriate DOPA grafting density of DEX‐g‐DOPA, ultrahigh molecular weight of DNA were critical for the hydrogel formation (Figure S5, Supporting Information). DEX‐g‐DOPA with different molecular weights (6k, 10k, and 40k Da) were all able to interact with DNA to form the hydrogel. The G′ value of hydrogel was increased from 60 to 220 Pa with the increase of the molecular weights of DEX‐g‐DOPA (Figure S5A, Supporting Information).

The molecular mechanism for the formation of hydrogel was discussed in detail (Discussion S2, Supporting Information). Fluorescence spectra results of ethidium bromide (EtBr, 20 × 10^−3^ m) in DNA/DEX‐g‐DOPA mixed solution at 25, 90, and after cooling back to 25 °C indicated that DEX‐g‐DOPA interfered the repairing of hydrophobic DNA bases during the annealing process (Figure S6, Supporting Information). Time‐dependent UV–vis spectra of DNA/DEX‐g‐DOPA mixed solution confirmed the phase separation of DNA induced by DEX‐g‐DOPA, as revealed by the appearance of scattering as the time elongated (Figure S7A–C, Supporting Information).^[^ ^30^ ^]^ In addition, the increase of absorption peak intensity at 324 nm along with time indicated the occurrence of DOPA oxidation and covalent crosslinking.^[^ ^31^ ^]^ Differential scanning calorimeter curves gave direct evidence of the presence of hydrophobic separated DNA bases in the hydrogel (Figure S7D, Supporting Information). Density functional theory results further suggested that DEX‐g‐DOPA mainly interacted with adenine (dA) and guanine (dG) groups of DNA bases, which were responsible for the phase separation of DNA (Figure S8, Supporting Information).^[^ ^30^ ^]^ UV–vis spectrum of DNA/DEX‐g‐DOPA mixed solution and X‐ray photoelectron spectroscopy (XPS) and FT‐IR of the hydrogel demonstrated the presence of hydrogen bonds and π–π stacking between DEX‐g‐DOPA and DNA (Figure S9, Supporting Information). Therefore, synergic permanent and dynamic double networks were integrated in this hydrogel. DOPA–DOPA covalent interactions among DEX‐g‐DOPA provided permanent crosslinking for hydrogel framework; meanwhile hydrophobic interactions derived from separated DNA bases, and hydrogen bonds and π–π stacking between DNA and DEX‐g‐DOPA formed dynamic interactions.

**Figure 2:**
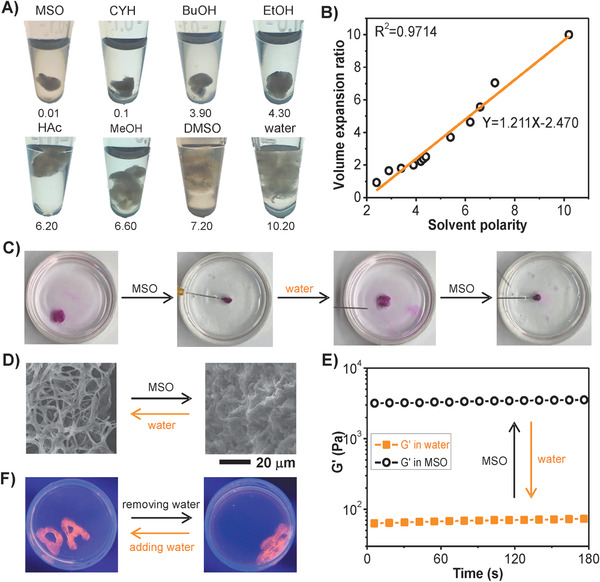
Volume‐ and shape‐responsiveness of DNA/DEX‐g‐DOPA hydrogel upon solvent polarity. A) Digital photos of the hydrogel immersed into different solvents. The attached number was polarity parameter of the corresponding solvents. MSO: petroleum ether; CYH: cyclohexane; BuOH: n‐butanol; EtOH: ethanol; HAc: acetic acid; MeOH: methanol; DMSO: dimethyl sulphoxide. B) The curve of volume expansion ratio for the hydrogel as a function of solvent polarity. C) Simultaneous volume and shape changes when the square‐shaped hydrogel was immersed into different solvents repeatedly. A square‐shaped hydrogel was first prepared in water. When soaked in MSO, the hydrogel was quickly aggregated into a dense form with volume shrinkage. When soaked in water again, the hydrogel returned to its birth shape with volume expansion reversibly. The hydrogel was stained with Rhodamine B. D) SEM images of the hydrogel immersed in water and MSO. Accompany with the exchange of solvents, the micromorphology of nanofibers was changed from loose structures to compact structures reversibly. E) The G′ value of the hydrogel immersed in water and MSO as a function of time. F) Water‐triggered volume and shape changes of the hydrogel. Hydrogels with the shapes of letters A and D were first prepared in water. After removing water, the hydrogels were aggregated with volume shrinkage. When water was reintroduced, the hydrogels could return to their birth shapes quickly. The hydrogels were stained with GelRed.

We explored the volume‐ and shape‐responsiveness of the hydrogel upon solvent polarity. First, the volume of hydrogel increased gradually with the increase of solvent polarity (**Figure** **2**A). The hydrogel was highly expanded in polar solvents, such as water and dimethyl sulphoxide, and shrank in nonpolar solvents, such as petroleum ether and cyclohexane. Remarkably, the volume expansion ratio of hydrogel showed a linear correlation with the solvent polarity when the value of solvent polarity was higher than 2, which provided a simple way to directly distinguish different solvents (Figure 2B). Furthermore, the G′ value of the hydrogel could be precisely controlled by immersing the hydrogel into different solvents (Figure S10, Supporting Information). With the increase of solvent polarity from 0.01 to 10.2, the G′ value of hydrogel gradually increased from 59 to 3651 Pa (Figure S11, Supporting Information).

To further investigate the effects of solvent polarity on volume, micromorphology and G′ value of hydrogel, water and petroleum ether (MSO) were utilized for the demonstration. Specifically, a square‐shaped hydrogel was first prepared in water using the square‐shaped mold. When soaked in MSO, the hydrogel was quickly aggregated into a dense form with volume shrinkage within a few seconds. When soaked in water again, the hydrogel returned to its birth shape with volume expansion reversibly (Figure 2C). Although the hydrogel was super‐soft, the hydrogel still maintained the original shape (square) even though undergoing the repeated cycles of replacing the solvents. Accompany with the exchange of solvents, the micromorphology of nanofibers was changed from loose structures to compact structures reversibly (Figure 2D). In MSO, nanofibers were aggregated to drive the volume shrinkage of hydrogel, and the hydrophobic interactions between nanofibers and nonpolar solvents were responsible for this process.^[^ ^28^ ^]^ In this situation, the micromorphology of nanofibers was coiled, aggregated, and compact. In water, the nanofibers were unwrapped to drive the volume expansion of hydrogel, and thus the micromorphology was stretched, intertwined, and loose. Hydrogen bonds between DNA/DEX‐g‐DOPA and polar solvents accelerated this process. Moreover, the modulus of hydrogel changed accordingly in water and MSO. In water, the G′ value of hydrogel was ≈59 Pa. When the hydrogel was immersed in MSO, the G′ value (3651 Pa) increased nearly 62 times than that in water. During this process, hydrophobic interactions between the nanofibers and MSO molecules were significantly enhanced and thus resulting in the formation of the compact nanofibrous structures (Figure 2E). Moreover, the hydrogen bonds existing in the hydrogel were significantly weakened in MSO, and thus the hydrophobic interactions were the main driving force for the volume shrinkage of the hydrogel.

Besides, the hydrogel showed super‐fast volume‐ and shape‐responsiveness upon water. The shapeless hydrogels with volume shrinkage recovered their birth shapes with volume expansion quickly by reintroducing water. To investigate this property, we first prepared hydrogels in water using molds with the shapes of letters A and D. After removing water of hydrogels, A‐ and D‐shaped hydrogels were aggregated with the volume shrinkage. When water was reintroduced, the hydrogels could return to their birth shapes quickly (Figure 2F).

**Figure 3:**
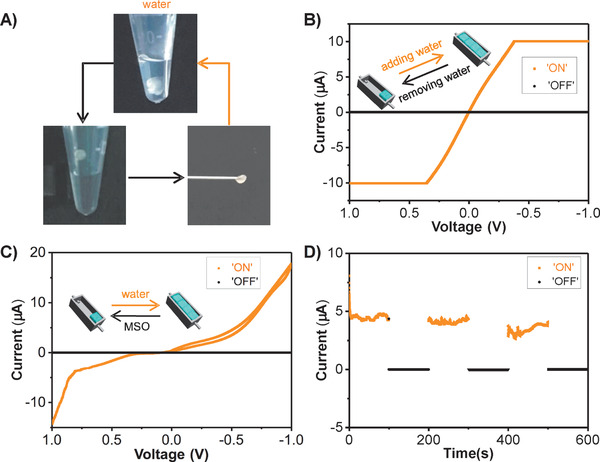
Electric circuits switched by water and water/MSO switch pair using DNA/DEX‐g‐DOPA hydrogel as dynamic wires. A) Digital photos of the hydrogel with volumetric responsiveness upon water. When the hydrogel was taken out from water, the volume of hydrogel was only one‐tenth of that in water within 1 s. When soaked in water again, the hydrogel returned to its birth volume. B) Cyclic voltage–current curve of the electric circuit switched by adding/removing water. C) Cyclic voltage–current curve of the electric circuit that used water and MSO as the switch pair. D) Current‐time curve of the electric circuit by replacing the solvents (MSO and water) repeatedly, demonstrating the stability of the electric circuit.

As a demonstration of application, a serials of dynamic hydrogel‐based electric circuits were fabricated. First, the electric circuit including a bulb with unilateral conduction, variable voltage power supply, and DNA/DEX‐g‐DOPA hydrogel as dynamic wires was fabricated to be able to power a bulb, indicating the feasibility of the construction of hydrogel‐based electric circuits (Figure S12, Supporting Information). Notably, the hydrogel showed superfast volume‐responsiveness upon water. When the hydrogel was taken out from water, the volume of hydrogel was only one‐tenth of that in water within 1 s (**Figure** **3**A). When soaked in water again, the hydrogel returned to its birth volume. Hydrophobic interactions were mainly responsible for this process. When the hydrogel was taken out from water, the presence of hydrophobic interactions would repel excess water from the hydrogel.^[^ ^28^ ^]^ Based on this special property, the electric circuit that used DNA/DEX‐g‐DOPA hydrogel as dynamic wires was fabricated through adding/removing water (Figure 3B). When water was added into the conductive channel, the hydrogel with a very small volume was highly expanded and conformed to the shape of the channel linking the two electrodes together, thus turning on the circuit. By removing water of the channel, the hydrogel shrank quickly to shut off the current.

By virtue of the volumetric responsiveness of the hydrogel upon different solvents, the electric circuit that used water and MSO as the switch pair was fabricated, in which DNA/DEX‐g‐DOPA hydrogel was served as dynamic wires (Figure 3C). Gold nanoparticles were doped into the hydrogel to enhance the conductivity of the hydrogel. When MSO was added into the channel, the hydrogel was aggregated with volume shrinkage, and thus the electric circuit was shut off. When MSO was replaced by water, the volume expansion of the hydrogel occurred and the electric circuit turned on again. Due to the large difference of solvent polarity between water and MSO, low amounts of solvents were enough to switch the electric circuit. The robust stability of electric circuit was demonstrated as revealed by the cyclic current‐time curve (Figure 3D). The water/MSO cycle was repeated three times, and the current value was still relatively stable every time as the electric circuit was triggered on/off. Other solvent pairs with different polarities could be employed as switch pairs to trigger on/off of the electric circuit.

**Figure 4:**
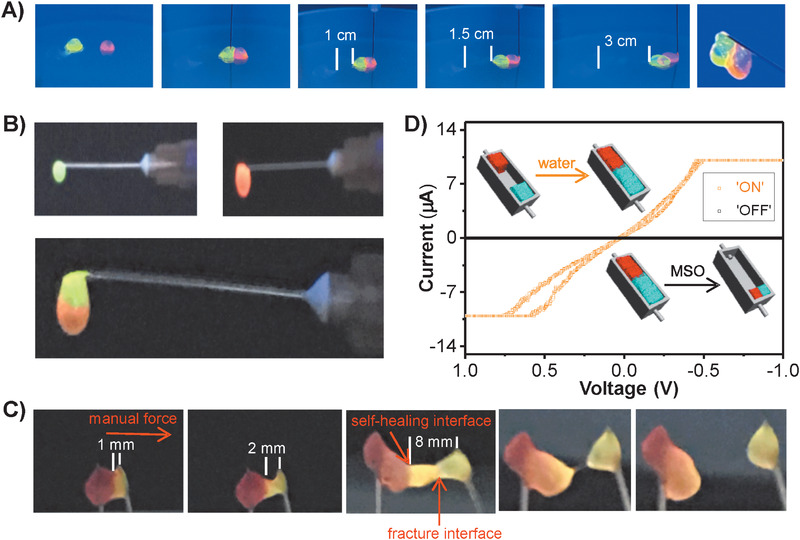
Electric circuit switched by the water/MSO switch pair using the self‐healed DNA/DEX‐g‐DOPA hydrogel as dynamic wires. A) Self‐healing of the two pieces of rectangle‐shaped hydrogels. One integrated hydrogel was cut into two pieces of rectangle‐shaped hydrogels and then the two pieces self‐healed for 20 s to achieve the cooperative motions of the self‐healed hydrogel. The hydrogel was able to be hung in the air. Two pieces of hydrogels were stained with SYBR Green I (green) and GelRed (red), respectively. B,C) Manual tensile test of the self‐healed hydrogel. Two pieces of hydrogels stained with red and green were held together to form an integrated hydrogel. Manual force was then applied to the right side of the self‐healed hydrogel. The initial length of green hydrogel was 1 mm. When the manual force increased gradually, the length of green hydrogel increased to 8 mm without fracture at the self‐healing interface, further demonstrating the self‐healing of the hydrogel. D) Cyclic voltage–current curve of the electric circuit that used the self‐heald hydrogel as dynamic wires.

The electric circuit switched by the water/MSO switch pair was fabricated successfully using the self‐healing DNA/DEX‐g‐DOPA hydrogel as dynamic wires. To visually demonstrate the self‐healing property of hydrogel, one integrated hydrogel was cut into two pieces with rectangle shapes, in which one piece was stained with SYBR Green I (green) and the other piece was stained with GelRed (red) (**Figure** **4**A). When two pieces of hydrogels were held together for 20 s, they formed an integrated hydrogel. It was inferred that motions and re‐entanglements of DNA/DEX‐g‐DOPA nanofibers driven by dynamic interactions at interface were responsible for the self‐healing property. The manual force was applied onto the right side of red hydrogel, resulting in the cooperative motions of the self‐healed hydrogel without the separation of the two pieces. Finally, the self‐healed hydrogel with original rectangle shapes could be hung in the air, visually demonstrating the self‐healing performance of hydrogel.

In addition, a manual tensile test was conducted to further confirm the self‐healing performance of hydrogel. Two pieces of hydrogels stained with red and green were held together, they could form an integrated hydrogel in the air (Figure 4B). Manual force was then applied to the right side of the self‐healed hydrogel (Figure 4C). The initial length of green hydrogel was 1 mm. When the manual force increased gradually, the length of green hydrogel increased to 8 mm without fracture at the self‐healing interface. Alternatively, an obvious fracture interface appeared inside the green hydrogel. Finally, the fracture of hydrogel occurred by further increasing the manual force. It was apparent that the fracture interface was not the self‐healing interface, further confirming that the interface between red and green hydrogel was completely self‐healed. By virtue of the self‐healing performance and solvent‐triggered volume responsiveness of the hydrogel, a self‐healing hydrogel‐based electric circuit that used water and MSO as the switch pair was designed (Figure 4D). First, two pieces of the hydrogels were placed in the both sides of the channel. The two pieces were not able to contact with each other and thus the electric circuit was cut off. When small amount of water was added into the channel, volume expansion of the two pieces occurred and then the two pieces contacted with each other to connect two electrodes together, turning on the electric circuit. By replacing water with MSO, the self‐healed hydrogel shrank quickly and was located at the one side of the channel to cut off the electric circuit.

**Figure 5:**
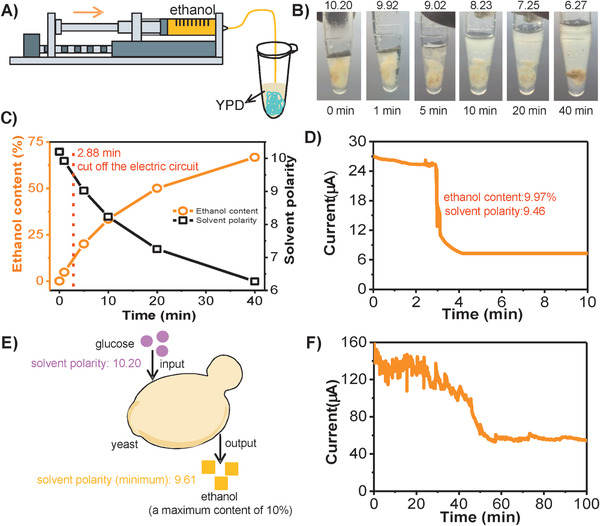
Electric circuits switched by a microbial metabolism process which produced ethanol using DNA/DEX‐g‐DOPA hydrogel as dynamic wires. A) Scheme for the process of ethanol injection into yeast extract peptone dextrose medium (YPD) containing DNA/DEX‐g‐DOPA hydrogel. The injection rate was 5 µL min^−1^. B) Digital photos of the hydrogel with the gradually decreased volume during the process of ethanol injection. C) Ethanol content and solvent polarity in YPD as a function of the time in (B). With the increase of ethanol content, the polarity in YPD decreased gradually, thus resulting in the volume shrinkage of the hydrogel. D) Current‐time curve of the electric circuit switched by the process of ethanol injection. At the beginning, the current values were stable; after 2.88 min injection of ethanol, the volume of hydrogel decreased significantly and the separation between hydrogel and the electrodes occurred, resulting in the remarkable decrease of the current value. Notably, when the electric circuit was cut off at 2.88 min, the ethanol content was 9.97% and the solvent polarity was only 9.46. E) Scheme for the process of yeast fermentation. During the process, the maximum production of ethanol was 10% in theory and thus the minimum polarity of the system was only 9.61. F) Current‐time curve of the electric circuit switched by a microbial metabolism process which produced ethanol. Along with the constant accumulation of ethanol, a significant reduction of current value occurred accompanied with the separation between hydrogel and the electrodes.

Taking full advantages of solvent‐triggered volume responsiveness and high biocompatibility of the hydrogel, a living system that produced solvents showed great potential to dynamically trigger the volume change of the hydrogel. As a proof‐of‐concept demonstration, yeast continuously produced ethanol through a microbial fermentation process, which was thus employed to design the microbial fermentation process triggering electric circuit that used DNA/DEX‐g‐DOPA hydrogel as a dynamic wire. Along with the consumption of glucose, the content of ethanol gradually increased during the fermentation process. This process provided dynamic and changeable solvent polarity to trigger off the electric circuit. First, a microinjection pump was used to mimic the fermentation process of yeast, which continuously injected ethanol into yeast extract peptone dextrose medium (YPD) (**Figure** **5**A). With the increase of ethanol content, the polarity of YPD decreased gradually, thus resulting in the volume shrinkage of the hydrogel (Figure 5B). Specifically, when the injected time increased from 0 to 40 min, the ethanol content was increased from 0% to 66.67% and the solvent polarity of YPD would decreased from 10.20 to 6.27 accordingly (Figure 5C). An electric circuit switched by the process of continuously injecting ethanol was fabricated. Real‐time current‐time curve was measured to reflect dynamic changing process of the volume of hydrogel (Figure 5D). At the beginning, the current values were stable; after 2.88 min injection of ethanol, the volume of hydrogel decreased significantly and the separation between the hydrogel and electrodes occurred, resulting in the remarkable decrease of the current value. Notably, when the electric circuit was cut off at 2.88 min, the ethanol content was only 9.97% and the solvent polarity was 9.46.

A dynamic electric circuit switched by a microbial metabolism process producing ethanol was created successfully. During the microbial metabolism process, the maximum production of ethanol was 10% in theory and thus the change of the solvent polarity was 0.59 at most (Figure 5E). Therefore, our prepared hydrogel with super‐fast volume‐responsiveness with high sensitivity upon the change of solvent polarity was utilized to dynamically switch the electric circuit. Specifically, yeasts were first precultured in YPD for 2 h to enhance the metabolic activity, and then the hydrogel was immersed into YPD culture medium. With the consumption of glucose, yeasts produced ethanol, and the real‐time current‐time curve was measured. The current value was slightly unstable at the beginning, probably due to the perturbation of the fermentation process (Figure 5F). Along with the constant accumulation of ethanol, a significant reduction of current value occurred accompanied with the separation between the hydrogel and electrodes. Finally, the current value was relatively stable and low, which was from the poor conductivity of the culture medium. We emphasized that this is the first time to construct a dynamic electric circuit switched by a microbial metabolism process without human interventions, showing great potential for the remote and programmable control of the circuits.

Other applications including regulating cell adhesion and controlled drug delivery were demonstrated to illustrate the wide potential of our prepared hydrogel. The hydrogel with different concentrations (0.05, 0.1, 0.5, 1, and 5 mg mL^−1^) showed high cell viability to human cervical cancer cells (Hela cells) and smooth muscle cells (SMCs cells), thus confirming the high biocompatibility of the hydrogel (Figures S13–S15, Supporting Information). When SMCs cells were cocultured with the hydrogel, SMCs cells were able to be adhered onto the surface of nanofiber‐assembled hydrogel (Figure S16A–C, Supporting Information). With the prolongation of cell culture time, the number of SMCs cells adhered onto the surface of hydrogel gradually increased, showing excellent cell affinity and adhesion (Figure S16D, Supporting Information). The hydrogel was also served as a biomaterial for controlled drug release using DNase I as a trigger. With the increment of DNase I concentration, the rate of drug release was accelerated, indicating a controlled and enzyme‐responsive drug release manner (Figure S17E, Supporting Information).

## Conclusion

In summary, a super‐soft and dynamic DNA/DEX‐g‐DOPA hydrogel with super‐fast volume‐responsiveness and high sensitivity upon solvent polarity was designed and prepared. Due to the super‐soft mechanical strength of the hydrogel, the volume and shape of hydrogel changed within a few seconds by replacing solvents. The hydrogel responded sensitively to the change of solvent polarity. Although the change of solvent polarity was only 0.4, the volume change of hydrogel was visually distinguished. This special property allowed the construction of a serials of dynamic hydrogel‐based electric circuits using solvents as switches. Remarkably, microorganisms were able to produce solvents through the metabolism process, thus providing a dynamic trigger to drive the volume change of our prepared hydrogel. The electric circuit switched by the microbial metabolism process was achieved successfully. We envision that our hydrogel will be a potential candidate for the construction of smart dynamic systems in response to biological process, and the electric circuits will be highly promising in wide applications, such as smart medicine, soft robots, controlled electro‐catalysis, and flexible electronic devices.^[^ ^32^, ^33^, ^34^, ^35^, ^36^ ^]^

## Experimental Section

##### Materials

Dextran (DEX, average *M*_w_ = 6000 g mol^−1^, average *M*_w_ = 10 000 g mol^−1^, and average *M*_w_ = 40 000 g mol^−1^) (from Meryer Chemical Technology Co. Ltd), anhydrous dimethyl sulfoxide (DMSO, from Aladdin Chemical Technology Co. Ltd), 1,1’‐carbonyldiimidazole (CDI, from Meryer Chemical Technology Co. Ltd), and dopamine hydrochloride (DOPA, from Meryer Chemical Technology Co. Ltd) were used for synthesizing dopamine‐grafted dextran (DEX‐g‐DOPA). Salmon sperm DNA (DNA, ≈20 000 base pairs) (from Sigma‐Aldrich) was utilized for fabricating DNA hydrogel. Sodium periodate (from Yuanli Chemical Co. Ltd) was used for synthesizing oxidized dextran. 2,4‐Dinitrophenylhydrazine (DNPH, from Macklin Chemical Technology Co. Ltd) was used for determination of the oxidation degree of dextran. DNA size markers, GelRed nucleic acid dyestuff, SYBR Green I nucleic acid dyestuff, and DNase I were obtained from TIANGEN BIOTECH. Dulbecco's modified eagle high‐glucose medium (DMEM/High Glucose) and fetal bovine serum were purchased from HyClone. Glutaraldehyde (EM Grade), propidium iodide (PI), and calcein AM were purchased from Solarbio. As a yeast extract peptone dextrose medium (YPD) for Angel yeast, a mixture of 20 g L^−1^ of peptone, 10 g L^−1^ yeast extract powder, and 140 g L^−1^ glucose was prepared.

##### Synthesis of DEX‐g‐DOPA

DEX (0.2 g) was dissolved in 5 mL anhydrous DMSO first. CDI (0.2303 g) dissolved in 2 mL anhydrous DMSO was transferred into the DEX solution and stirred for 30 min under room temperature. DOPA (0.1797 g, mole ratio of glucose unit of DEX and DOPA was ≈1.3:1) dissolved in 1 mL anhydrous DMSO was then transferred into the CDI‐activated DEX solution. The room‐temperature reaction was carried out with magnetic stirring (450 rpm) in the atmosphere of N_2_ for 12 h. After reaction, the solution was transferred into a dialysis membrane (*M*_W_ = 3500) and dialyzed for 24 h to remove unreacted reagents. The final products were freeze‐dried and stored in desiccator before use. ^1^H NMR (400 MHz, DMSO (d‐6), d/ppm): 2.88 (t, 2H, —NH—CH2—CH2‐); 3.23 (t, 2H, —NH—CH2—CH2); 3.52 (t, 1H, DEX C4‐H); 3.58 (t, 1H, DEX C2‐H); 3.71 (t, 1H, DEX C3‐H); 3.73–3.78 (q, 1H, DEX C5‐H); 3.89–4.01 (d, 1H, DEX C6‐H); 4.97 (d, 1H, DEX C1‐H); 6.75 (d, 1H, arom.); 6.85 (s, 1H, arom.), and 6.91 (d, 1H, arom.).

##### Preparation of DNA/DOPA Hydrogel

DNA was dissolved in deionized water with a concentration of 5 w/v% at higher temperature, and DEX‐g‐DOPA dissolved in deionized water with a concentration of 10 w/v% was added to the heated DNA solution. The volume ratio was 1:1. The hydrogel would form spontaneously in couple of days.

*Synthesis of Oxidized DEX*: 3.3 g of sodium periodate was transferred into 50 mL of 10 w/v% DEX solution (*M*_w_ = 40 000 g mol^−1^). The room‐temperature reaction was stirred for 1.5 h at dark conditions, after which the solution was transferred into a dialysis membrane (*M*_W_ = 12 000) and dialyzed for 3 d at 4 °C. The final products were freeze‐dried and stored in desiccator before use. ^1^H NMR analysis (400 MHz, D_2_O) was utilized to determine the structure of oxidized DEX.

##### Determination of Oxidation Degree of DEX

The oxidation degree of DEX was determined using a quantitative reaction of 2,4‐dinitrophenylhydrazine (DNPH). A solution of 4 w/v% oxidized DEX (250 µL) was mixed with 0.05 m DNPH (500 µL) (mixed solution of 1:1 v/v acetonitrile and 2 m HCl for dissolving DNPH) and incubated for 20 min under stirring. Then, the orange precipitate formed and the solution was extracted with 500 µL of ethyl acetate. The amount of unreacted DNPH could be calculated from the optical density at 350 nm using UV–vis spectra.

##### Fluorescence Microscopy Imaging

High resolution images of the hydrogel were obtained by fluorescence microscopy (Nikon). The hydrogel was stained with SYBR Green I or GelRed.

##### Rheology Test

The rheological properties were carried out on a HR‐2 rheometer (TA Instruments) equipped with a temperature controller. The test was performed in an 8 mm parallel‐plate geometry using 100 µL DNA/DOPA hydrogel. Temperature rheological test was carried out from 25 to 75 °C at a rate of 2 °C min^−1^ at fixed frequency (1 Hz) and strain (1%). Time scanning was performed with a fixed strain (1%) and a fixed frequency (1 Hz) at 25 °C for 3 min. Strain scanning was conducted from 0.1% to 1000% with a fixed frequency (1 Hz) at 25 °C.

##### SEM

All hydrogels were placed onto the sample stage using conductive adhesive. Then, the sample stage was placed in liquid nitrogen to achieve quick‐freeze. Finally, the samples were freeze‐dried and metal‐coated with Au for SEM (Hitachi‐S4800 FESEM).

##### XPS

XPS was performed on a VGESCALAB220i‐XL spectrometer equipped with a hemispherical analyzer. Survey (wide) and high‐resolution (narrow) scans were recorded. The base pressure in the analysis chamber was less than 8.0 × 10^−9^ mbar. All data were processed using Avantage software, and the energy calibration was referenced to the C 1s peak at 285.0 eV.

##### Volume Expansion Experiments

The hydrogel was immersed into different kinds of solvents with different solvent polarities. The volume of hydrogel immersed in each kind of solvent was measured and named as *V*_2_. The volume of hydrogel placed in the air was measured and named as *V*_1_. The Volume expansion ratio of the hydrogel in different solvents could be calculated using the following formula (1)

(1) $$\mathrm{Volume}\;\mathrm{expansion}\;\mathrm{ratio}=V_{2}/V_{1}$$

##### Electrical Property Measurement

To test the conductivity property of the hydrogel, a simple circuit was established, including a bulb with unilateral conduction, DNA/DOPA hydrogel and a variable voltage power supply. The voltage was changed from −5 to 5 V, followed by changed from 5 to −5 V. The current–voltage curves were measured using a Keithley 6430. To test the ability of the hydrogel as an electric circuit switch, hydrogel formed in a mold using petroleum ether as solvent was aggregated with smaller volume and the electric circuit was in the state of “OFF.” When petroleum ether was fully replaced by water, the hydrogel expanded and electric circuit was in the state of “ON.” The current–voltage curves were measured. The voltage changed from −1 to 1 V and the scan rate was set as 10 mV s^−1^. In order to prove the robustness of hydrogel‐based electric circuit, the current‐time curve was measured.

##### Hydrogel Circuits Switched by Yeast Fermentation

In order to mimic the process of ethanol production, microinjection pump was used to inject ethanol into yeast extract peptone dextrose medium (YPD). The injection rate was 5 µL min^−1^. The current‐time curve was measured for real time monitoring the process of yeast fermentation. The voltage was 0.5 V. Biocircuits switched by yeast fermentation were fabricated. Specifically, yeast were dispersed in YPD for culturing 2 h, then the hydrogel was immersed into the obtained culture medium for the construction of circuits. The current‐time curve was detected.

##### In Vitro Cell Culture

SMCs were used as the target cells for in vitro cell culture of DNA/DOPA hydrogel. Before cell seeding, the hydrogel was purified in PBS, followed by sterilized by 75% ethanol for 24 h. The obtained hydrogel was placed in a 96‐well plate with DMEM and swelled to the equilibrium state for 2 days at 37 °C. Cells were seeded on hydrogels and left undisturbed in an incubator for 3 h to allow for cell attachment. After 1, 3, and 5 days of culture, the cells grown on the hydrogel were stained with calcein AM, and observed by fluorescence microscope. The viability of cells grown on the hydrogels was analyzed by thiazolyl blue tetrazolium bromide MTT assay. After 1, 3, and 5 days culture, MTT solution was added to each well, and the plate was incubated at 37 °C for 4 h. After removing medium, the resulting purple formazan was dissolved in DMSO, and the absorbance at 490 nm was measured.

##### In Vitro Drug Release

DNA/DOPA hydrogel was expected as a controlled drug release system by using DNase I as a trigger. A unique advantage was that both the building blocks (DNA) and the fibrous hydrogel itself (physical enclosure, high specific surface area of DNA nanofibers) were used as drug reservoirs. Hydrogel with doxorubicin was loaded. DNase I with 50 and 500 U mL^−1^ were utilized.

## Conflict of Interest

The authors declare no conflict of interest.

## Supporting information

Supporting Information
