# Supplementary material for: Super‐Soft DNA/Dopamine‐Grafted‐Dextran Hydrogel as Dynamic Wire for Electric Circuits Switched by a Microbial Metabolism Process
Source: Adv Sci (Weinh). 2020 May 25;7(13):2000684. doi: 10.1002/advs.202000684 (PMC7341087; doi:10.1002/advs.202000684)
Supplement: Supplementary file 1 — Supporting Information [file ADVS-7-2000684-s001.pdf]

Copyright WILEY-VCH Verlag GmbH & Co. KGaA, 69469 Weinheim, Germany, 2016.

Supporting Information

Super-soft DNA/Dopamine-grafted-dextran Hydrogel as Dynamic Wire for Electric Circuits Switched by a Microbial Metabolism Process

Jinpeng Han[1], Yuchen Cui[1*], Xinpeng Han[1], Chenyu Liang[1], Wenguang Liu[2], Dan Luo[3], Dayong Yang[1]**

1: Frontier Science Center for Synthetic Biology, Key Laboratory of Systems Bioengineering (MOE), School of Chemical Engineering and Technology, Tianjin University, Tianjin, 300350, P.R. China
2: Tianjin Key Laboratory of Composite and Functional Materials, School of Materials Science and Engineering, Tianjin University, Tianjin 300350, P.R. China
3: Department of Biological & Environmental Engineering, Cornell University, Ithaca, New York 14853, United States
*Corresponding author: Professor Dayong Yang, E-mail: dayong.yang@tju.edu.cn

Keywords: DNA hydrogel, microbial metabolism, volume responsiveness, electric circuits, synthetic biology

Discussion S1: The influence factors for the formation of DNA/DEX-g-DOPA hydrogel

Discussion S2: The molecular mechanism for the formation of the hydrogel

Fig. S1: Characterization of DEX-g-DOPA

Fig. S2: Molecular weight of natural salmon sperm DNA

Fig. S3: Fluorescence images of the nanofiber-assembled hydrogel

Fig. S4: The effect of DOPA groups on the formation of hydrogel

Fig. S5: The effect of molecular weight on the formation of hydrogel

Fig. S6: Fluorescence spectra of EtBr in DNA/DEX-g-DOPA mixed solution

Fig. S7: UV-Vis spectra and DSC curves of DNA/DEX-g-DOPA mixed solution

Fig. S8: Density functional theory results between DOPA and DNA bases

Fig. S9: The spectral characterization of the hydrogel

Fig. S10: Rheological strain sweep of the hydrogel immersed in different solvents

Fig. S11: G' value of the hydrogel as a function of solvent polarity

Fig. S12: Cyclic voltage-current curve of the hydrogel based electric circuit

Fig. S13: Live/dead fluorescence images of Hela cells cultured with the hydrogel

Fig. S14: Live/dead fluorescence images of SMCs cells cultured with the hydrogel

Fig. S15: The cytotoxicity test of the hydrogel

Fig. S16: Cell adhesion and controlled drug release experiments of the hydrogel

Table S1. The summary and comparison of our prepared hydrogel with the previously-reported swelling materials.

Discussion S1: the influence factors of the formation of DNA/DOPA hydrogel

The presence of catechol groups, appropriate grafting density of DEX-g-DOPA, and ultra-high molecular weight and high concentration of DNA were critical for hydrogel formation. Oxidized dextran was synthesized via grafting aldehyde groups onto DEX backbone (**Fig. S4A**). ^{1}H NMR results confirmed the structure of oxidized DEX, and the grafting rate was approximately 12.9% (**Fig. S4B and C**). As expected, only DEX-g-DOPA (grafting rate 11.1%) was utilized for hydrogel formation among DEX, oxidized DEX and DEX-g-DOPA (**Fig. S4D**). The grafting rate of DEX-g-DOPA was adjusted by changing the mole ratio of DEX and DOPA. When the mole ratio was 1.3:1, the grafting rate was approximately 11.1% for hydrogel formation (**Fig. S1C**). When the mole ratio was 3:1, the grafting rate was only 5.2% and the hydrogel was not obtained due to the limited density of catechol groups (**Fig. S1D**). When the mole ratio was 1:3, DOPA was apt to form polydopamine driven by $\pi-\pi$ interactions, thus leading to the products insoluble in water. The high molecular weight of polymer was favor to its phase separation and further forming polymer-based hydrogel networks. Considering the molecular weight of DEX, DEX-g-DOPA with different molecular weights (6k, 10k and 40k Da) were all able to prepare the hydrogel. The G' value was changed from 60 to 220 Pa with the increment of the molecular weights of DEX-g-DOPA (**Fig. S5A**). In regard to the molecular weights of DNA, the molecular weights of DNA decreased gradually by increasing the treatment time of DNase I (**Fig. S5B**). However, only DNA without treatments of DNase I was used for hydrogel formation (**Fig. S5C**). According to the model of canonical ensemble[3], high DNA concentration was favor to forming bundle even fibrous structures, while low DNA concentration was favor to forming toroidal structures. Thus, DNA concentration was optimized as 5 w/v% (high concentration) for preparing nanofiber-assembled DNA/DOPA hydrogel.

Discussion S2: The molecular mechanism for the formation of the hydrogel

DNA composed of four deoxyribonucleotide monomers was regarded as a block copolymer, wherein the hydrophobic DNA bases were embedded into the interior structures of duplex DNA copolymers through Watson-Crick base-paring. When DNA solution was heated to a higher temperature (90 ^{o}C), the unwinding of duplex DNA occurred (**Fig. S6A**). After cooling back to 25 ^{o}C, the recovery of duplex DNA occurred, as demonstrated by the fluorescence spectra of ethidium bromide (EtBr) in DNA solution (**Fig. S6B**). When DNA was mixed with DEX-g-DOPA, DEX-g-DOPA could interfere the re-pairing of hydrophobic DNA bases during the annealing process, as indicated by the fluorescence spectra of EtBr in DNA/DEX-g-DOPA mixed solution at 25, 90 and after cooling back to 25 °C (**Fig. S6C and S6D**). Time-dependent UV–Vis spectra of DEX-g-DOPA/DNA mixed solution confirmed the phase separation of DNA induced by DEX-g-DOPA, as revealed by the appearance of scattering as the time elongated (**Fig. S7A**).[1] In contrast, phase separation was not observed in pure DNA and DEX-g-DOPA solution (**Fig. S7B and S7C**). Differential scanning calorimeter (DSC) thermograms gave direct evidence of unwinding of duplex DNA (**Fig. S7D**). Two endothermic peaks appeared in DNA samples, where M_1 indicated the presence of small amounts of DNA fragments owing to the unwinding of duplex DNA, and M_2 suggested the double helix structures of DNA.[2] After the introduction of DEX-g-DOPA, the peak intensity at N_1 was markedly enhanced, confirming that the phase separation of DNA occurred to form the separated DNA bases due to the enhanced interactions between DEX-g-DOPA and DNA bases. The new peak at M_3 suggested the enhanced thermal stability of the hydrogel, due to the formation DNA/DEX-g-DOPA nanofibers. Density functional theory results between DOPA and DNA bases indicated that DEX-g-DOPA mainly interacted with adenine (dA) and guanine (dG) groups of DNA bases, which were responsible for the phase seperation of DNA (**Fig. S8**). UV-Vis spectrum revealed the existence of intermolecular interactions between

DEX-g-DOPA and DNA (**Fig. S9A**). In detail, the absorption peaks at 260 and 277 nm were the characteristic peaks of DNA and DEX-g-DOPA, respectively. When DEX-g-DOPA was mixed with DNA, the absorption peak migrated to 274 nm, suggesting the enhanced interactions between DEX-g-DOPA and DNA. For a detailed analysis of intermolecular interactions, high-resolution X-ray photoelectron spectroscopy (XPS) was conducted. Elemental content results confirmed that all of C, N, O, and P elements existed in the hydrogel (**Fig. S9B**). The contents of N and P elements were 6.51% and 2.52%, respectively. The core-level C 1s spectrum was decomposed into three peaks, where C_I and C_{II} corresponded to C-H, C-N and C-O type carbon atoms, and C_{III} at 289.2 eV indicated the presence of π-π stacking (**Fig. S9C**). [3] The core-level N 1s spectrum was decomposed into two components, where N_I peak at 399.1 eV was attributed to the inter-strand base pairing of DNA, and N_{II} peak at 400.5 eV indicated the enhanced interactions between DEX-g-DOPA and DNA bases (**Fig. S9D**). [4] FT-IR results showed that the wavenumbers at 1238 and 1702 cm^{-1} of DNA were attributed to the antisymmetric vibration of phosphate groups and inter-strand base pairing, respectively (**Fig. S9E**).[5-6] When DNA was mixed with DEX-g-DOPA, the peak at 1702 cm^{-1} was narrowed down and shifted, further confirming the enhanced interactions between DEX-g-DOPA and DNA bases.[7] Combined with the core-level N 1s spectrum in **Fig. S9D**, it was inferred that the partial unwinding of duplex DNA occurred during the formation of the hydrogel. Besides, the peak at 1238 cm^{-1} was shifted to 1265 cm^{-1}, suggesting the enhanced hydrogen bonds between phosphate groups of DNA and DEX-g-DOPA.[5]

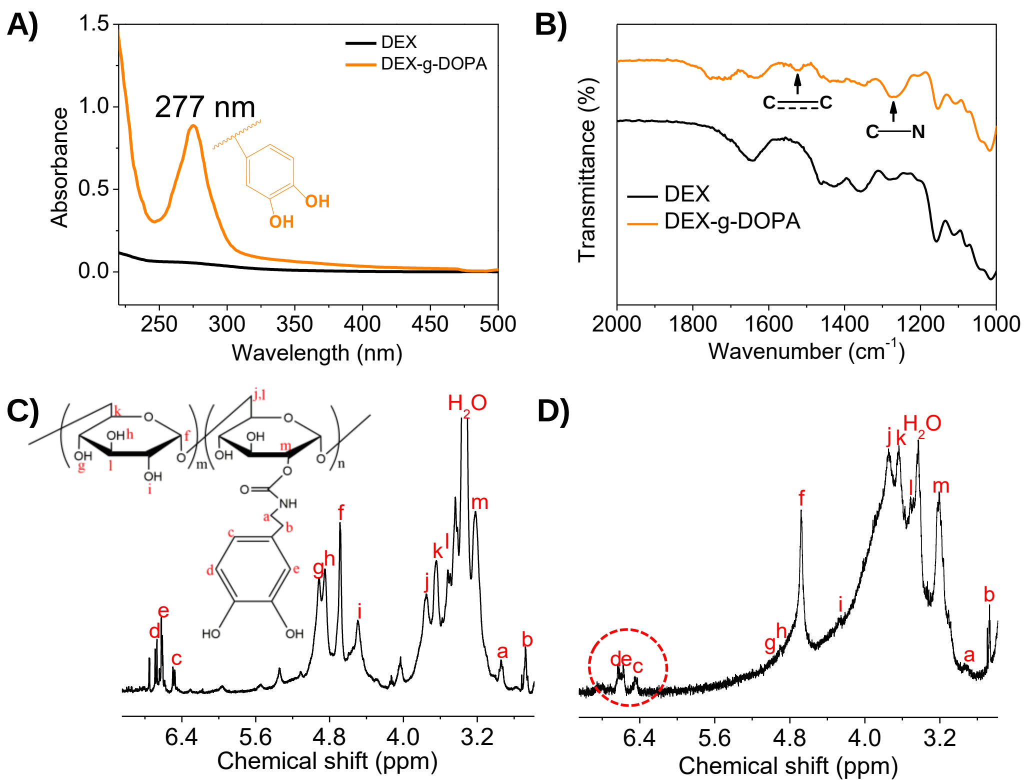

Figure S1. Characterization of DEX-g-DOPA. (A) UV–Vis spectra of DEX-g-DOPA and DEX. The strong absorption peak at 277 nm indicated the presence of catechol group, while the normal dextran had no significant absorption at 277 nm. **(B)** FT-IR spectra of DEX-g-DOPA and DEX. The presence of C–N bond at 1250 cm^{-1} and aromatic group at 1560 cm^{-1} indicated that DEX-g-DOPA was successfully synthesized. **(C)** ^{1}H NMR spectrum of DEX-g-DOPA. The grafting rate of DOPA was approximately 11.1% as calculated from the NMR spectrum. The mole ratio of glucose unit of DEX and DOPA was approximately 1.3:1. **(D)** ^{1}H NMR spectrum of DEX-g-DOPA. The grafting rate of DOPA was only 5.2%. The mole ratio of glucose unit of DEX and DOPA was approximately 3:1.

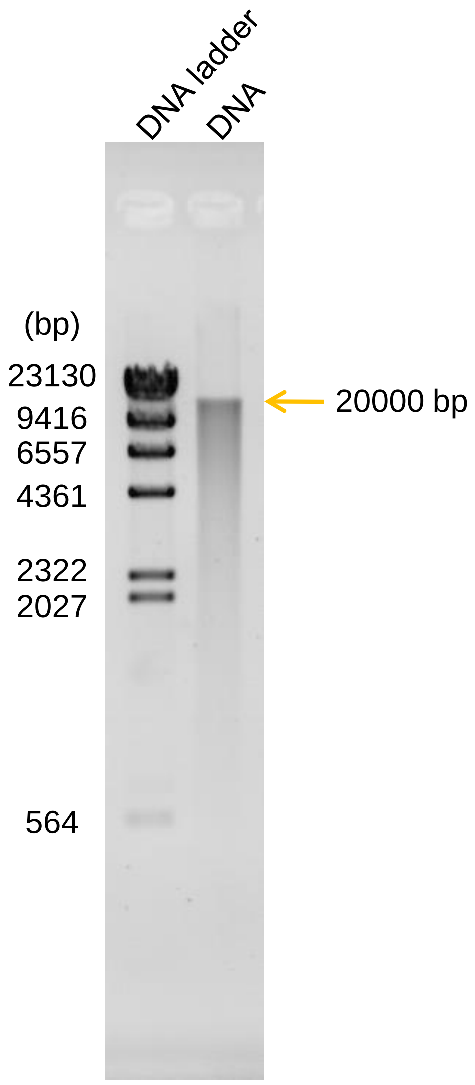

Figure S2. The 1% agarose gel electrophoresis of salmon sperm DNA. The DNA ladder was λ DNA/Hind III. The molecular weight of DNA was approximately 20 000 base pairs (12 million Da).

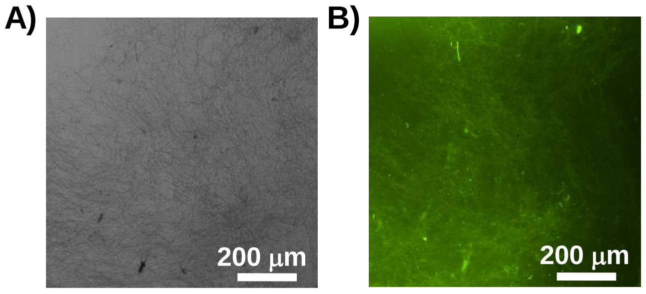

Figure S3. Fluorescence images of DNA/DEX-g-DOPA hydrogel under different magnifications. The hydrogel was composed of entangled nanofibers. The nanofibers were stained green by SYBR Green I, suggesting the formation of DNA-based nanofibers.

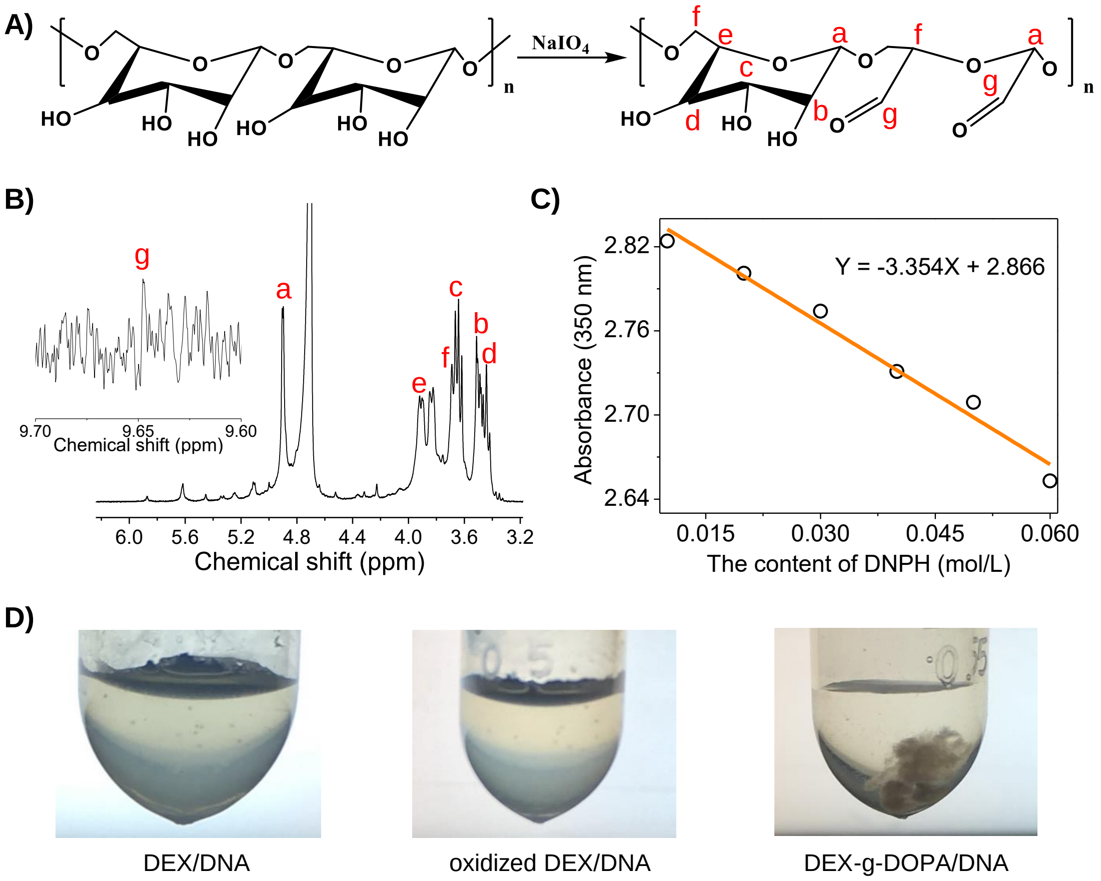

Figure S4. The effect of functional groups of DEX on the formation of DNA/DEX-g-DOPA hydrogel. (A) Scheme for the synthesis procedure of oxidized DEX. Sodium periodate was utilized as oxidant to synthesize oxidized DEX. **(B)** ^{1}H NMR spectrum of oxidized DEX. The protons of aldehyde groups was observed at δ 9.65 ppm with low intensity, indicating the aldehyde groups were partly conjugated to DEX. Also, the presence of several peaks between δ 5.8 and δ 4.2 ppm proved the formation of different hemiacetal structures in DEX. **(C)** The standard curve of 2,4-dinitrophenylhydrazine (DNPH). According to the standard curve, the oxidation degree of oxidized DEX was calculated as 12.9%. **(D)** Digital photos of DNA/DEX-g-DOPA hydrogel prepared using DEX grafted with different functional groups. Only DEX-g-DOPA was utilized for hydrogel formation, which demonstrated that the catechol groups was critical for preparing the hydrogel.

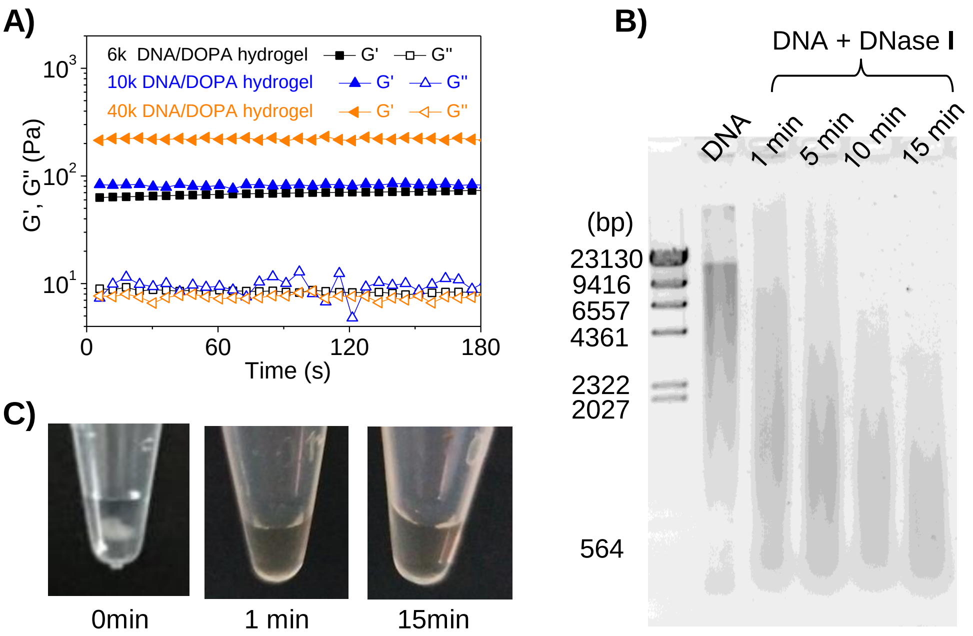

Figure S5. The effect of molecular weights of DEX-g-DOPA and DNA on the formation of DNA/DEX-g-DOPA hydrogel. (A) G’ and G’’ of the hydrogel as a function of time. Different molecular weights of DEX-g-DOPA (6k, 10k and 40k) were all utilized for preparing the hydrogel. G’ was increased from 60 to 220 Pa with the increment of molecular weight of DEX-g-DOPA. **(B)** The 1% agarose gel electrophoresis of DNA treated with DNase I for different times. The molecular weight of DNA was decreased gradually, as the treatment time of DNase I was ranged from 1 to 15 min. **(C)** Digital photos of the hydrogel prepared using DNA with different molecular weights. DEX (6k) was utilized for hydrogel formation. Only DNA without treatment of DNase I was used for hydrogel formation.

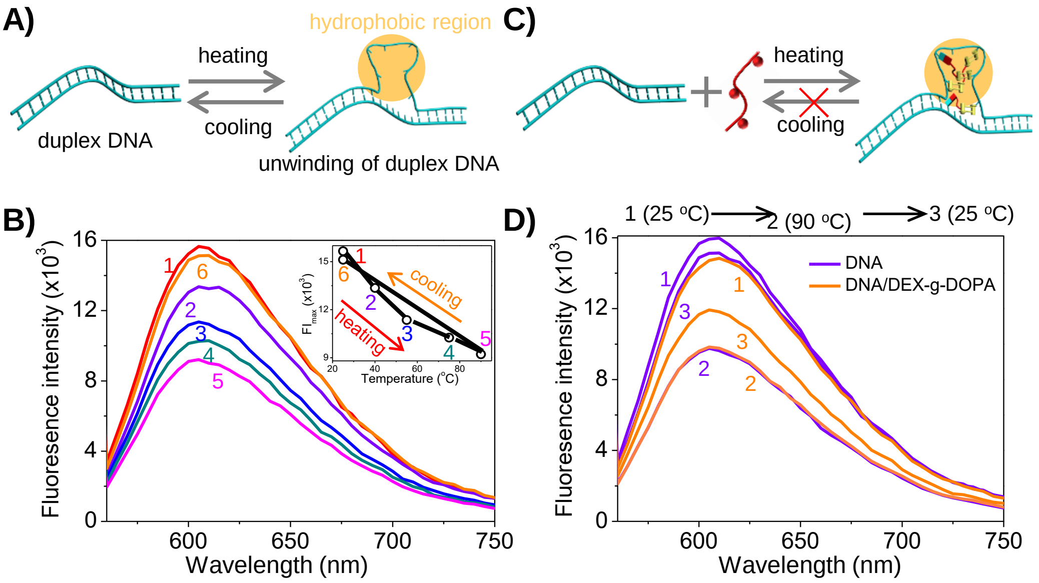

Figure S6. Fluorescence spectra of EtBr in DNA/DEX-g-DOPA mixed solution. (A and C) Scheme for the structural changes during the heating and cooling process. **A:** duplex DNA; **C:** DEX-g-DOPA/duplex DNA mixture. **(B)** Fluorescence spectra of ethidium bromide (EtBr, 20 μM) in duplex DNA solution (0.5 w/v%) at 25 (1), 40 (2), 55 (3), 75 (4), 90 (5) and after cooling back to 25 °C (6). The inset exhibited the fluorescence intensity of EtBr at 600 nm as a function of temperature. The numbers marked in the inset corresponded to those given in the spectra. **(D)** Fluorescence spectra of EtBr in DNA solution and DEX-g-DOPA/DNA mixed solution at 25 (1), 90 (2) and after cooling back to 25 °C (6).

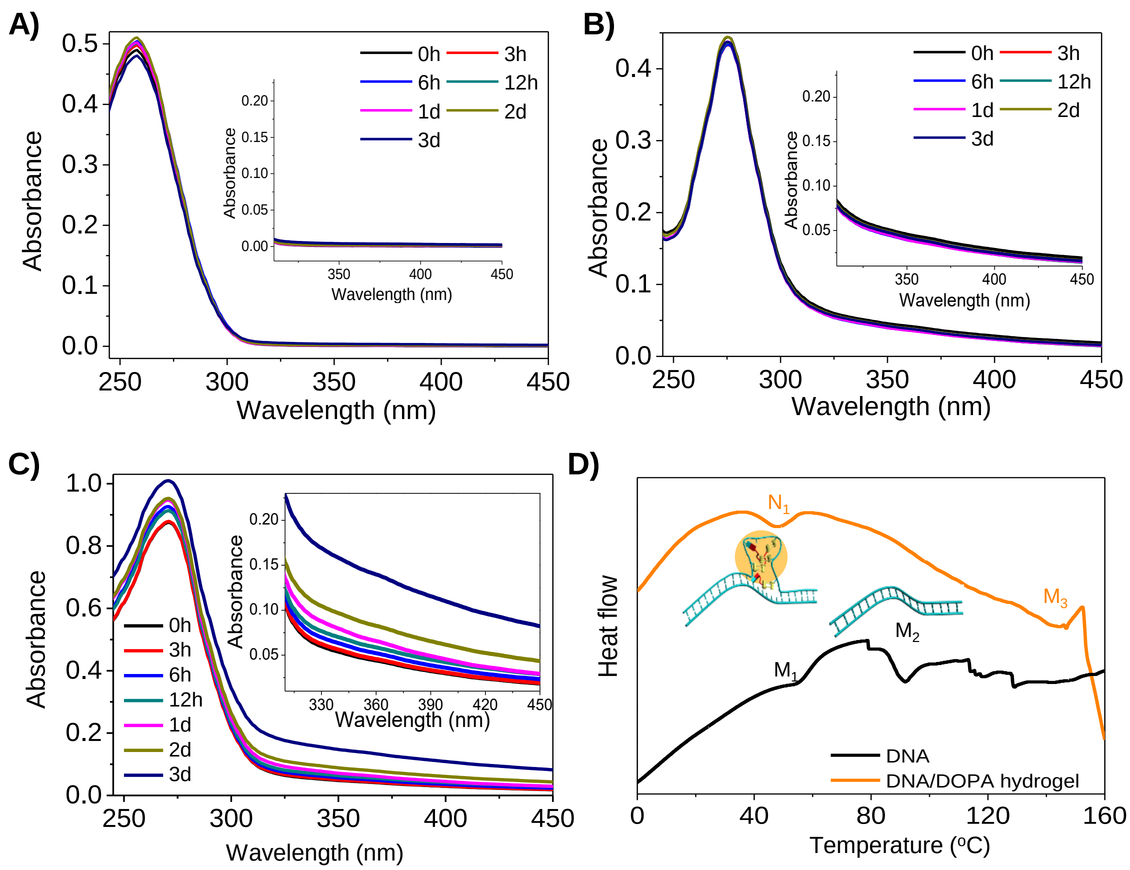

Figure S7. Time-dependent UV-Vis spectra and DSC curves of DNA/DEX-g-DOPA mixed solution. (A) Time-dependent UV–Vis spectra of DEX-g-DOPA/DNA mixed solution. **(B and C)** Time-dependent UV–vis spectra in pure DEX-g-DOPA and DNA solution. **(B)** DNA solution; **(C)** DEX-g-DOPA solution. Phase separation was not observed in pure DNA and DEX-g-DOPA solution. **(D)** DSC curves of DNA and the hydrogel.

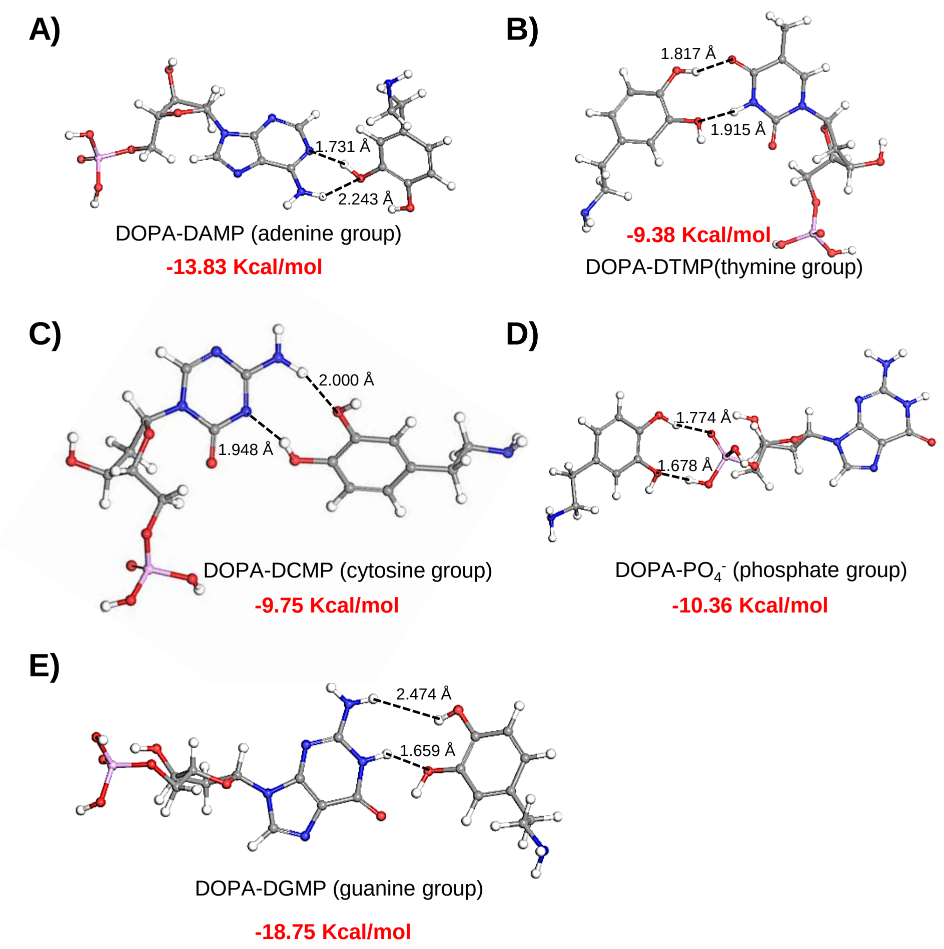

Figure S8. Density functional theory results between DOPA and different DNA bases.

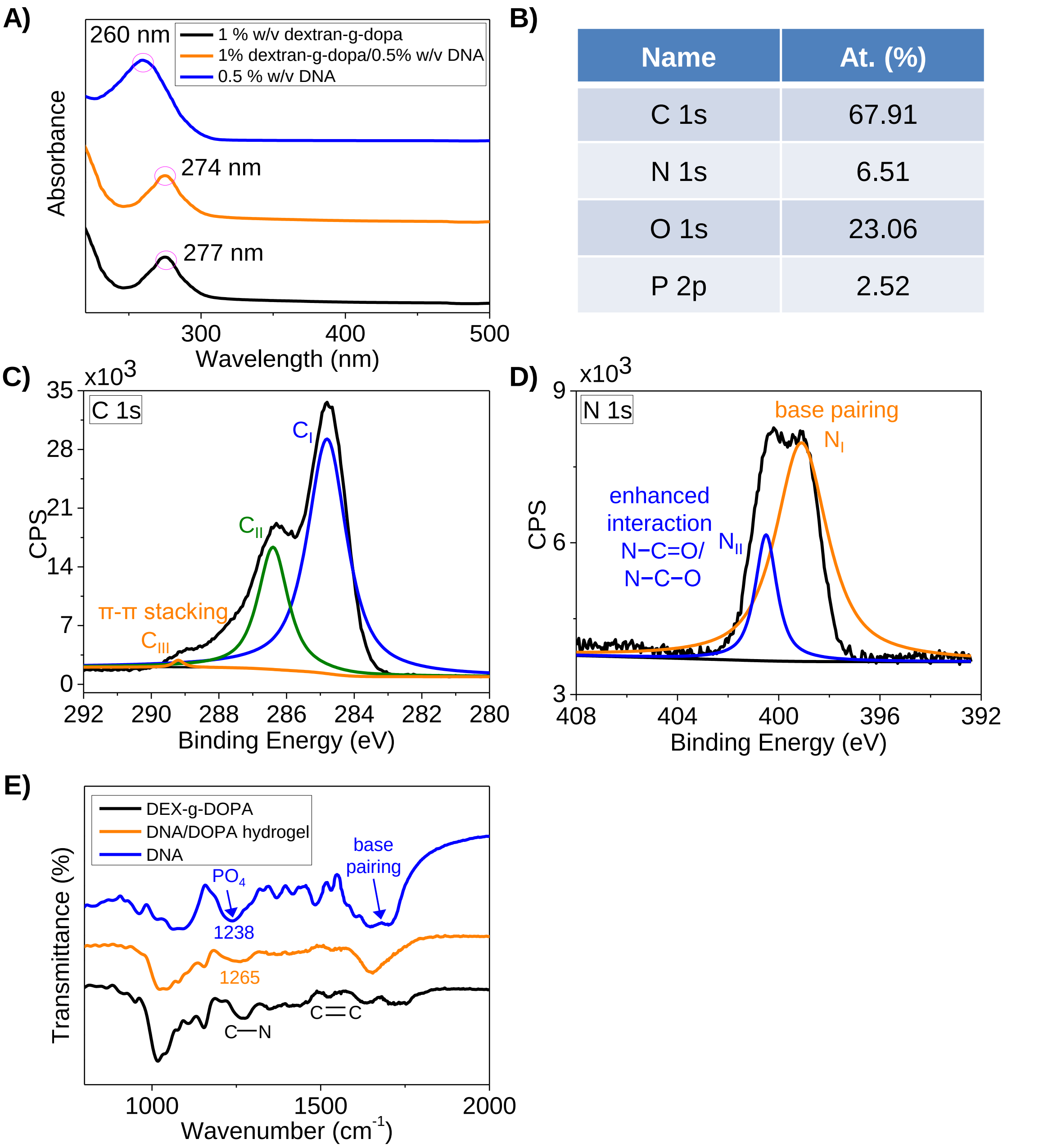

Name	At. (%)
C 1s	67.91
N 1s	6.51
O 1s	23.06
P 2p	2.52

Figure S9. The spectral characterization of the hydrogel. (A) UV-Vis curve of DEX-g-DOPA and DNA. Absorption peak at 260 nm was the characteristic peak of DNA. Absorption peak at 277 nm is the characteristic peak of catechol group of DEX-g-DOPA. When DEX-g-DOPA was mixed with DNA, the absorption peak was migrated, suggesting the interactions between DEX-g-DOPA and DNA. **(B)** Element contents of the dried gel. The contents of nitrogen and phosphorus element were 6.51% and 2.52%, respectively. **(C and D)** High-

resolution XPS of the gel. **(C)** The core-level C 1s spectrum; **(D)** The core-level N 1s spectrum. **(E)** FT-IR spectroscopy of DEX-g-DOPA, DNA and the hydrogel.

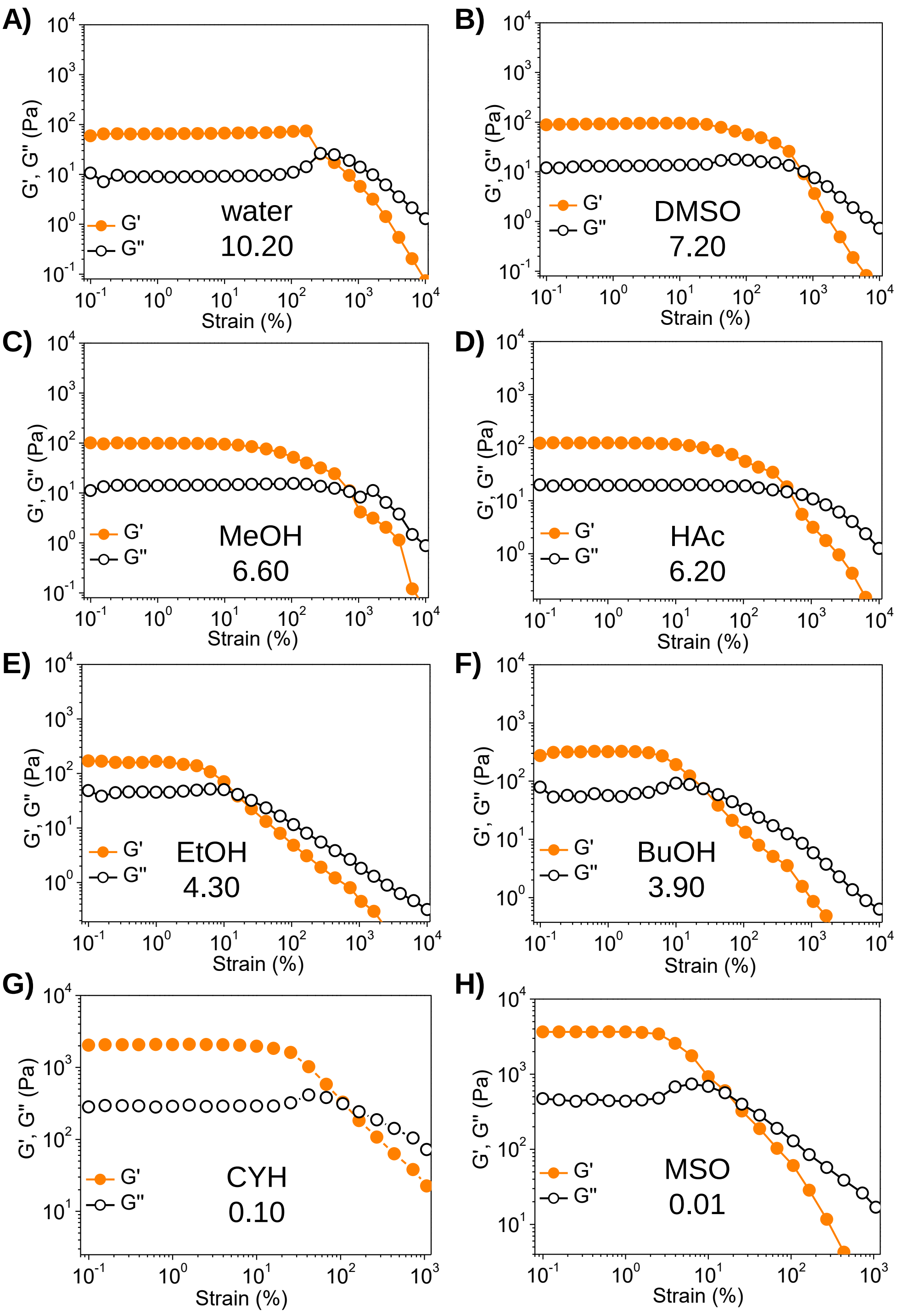

Figure S10. Rheological strain sweep of the hydrogel immersed in different solvents.

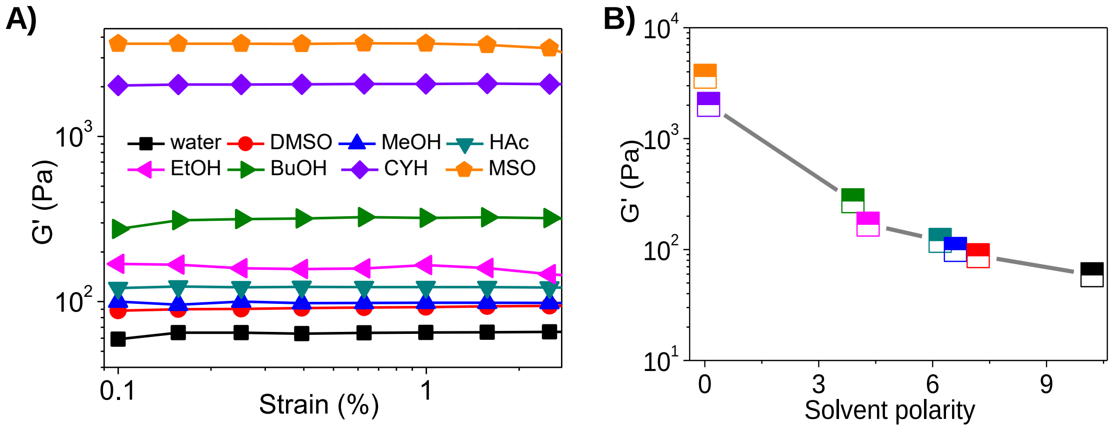

Figure S11. G' value of the hydrogel as a function of solvent polarity.

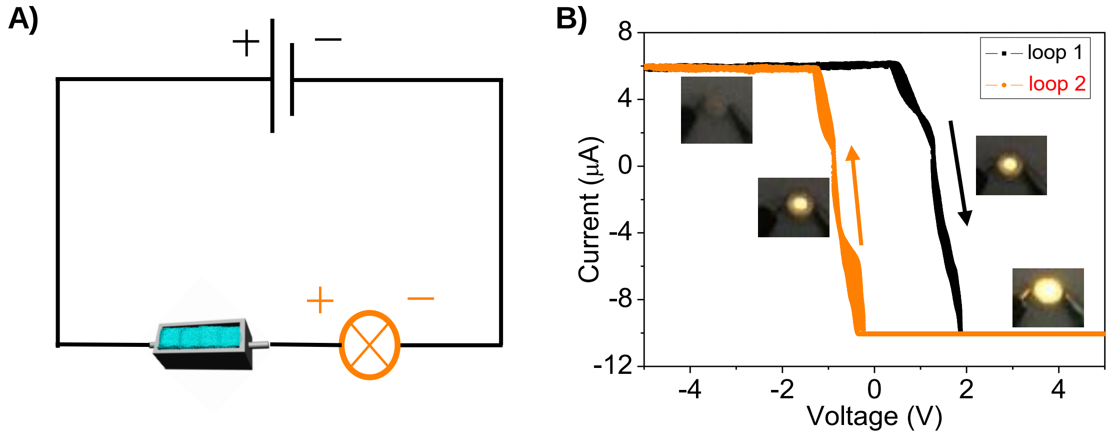

Figure S12. Cyclic voltage-current curve of the hydrogel based electric circuit.

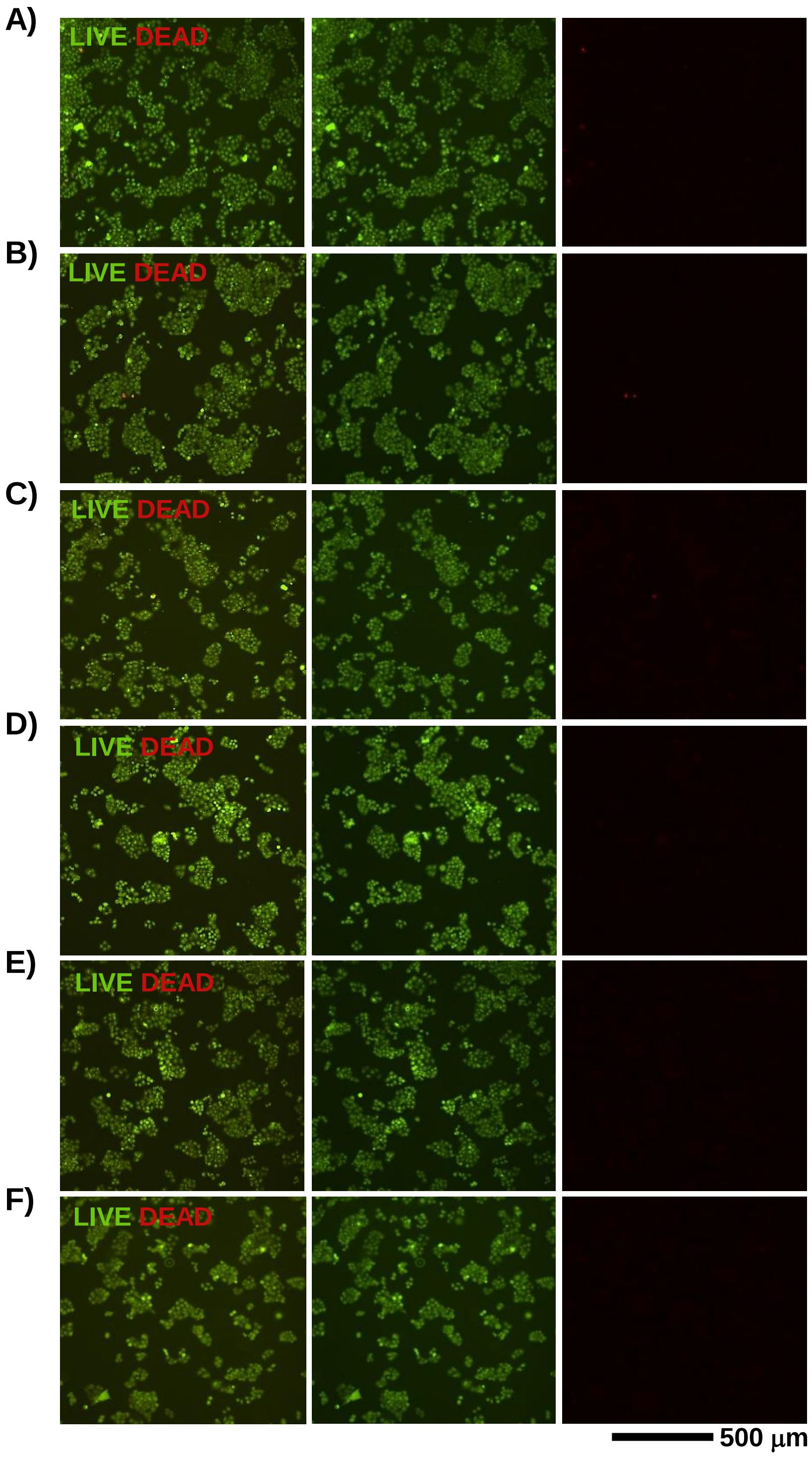

Figure S13. Representative live/dead fluorescence images of Hela cells cultured with the hydrogel.

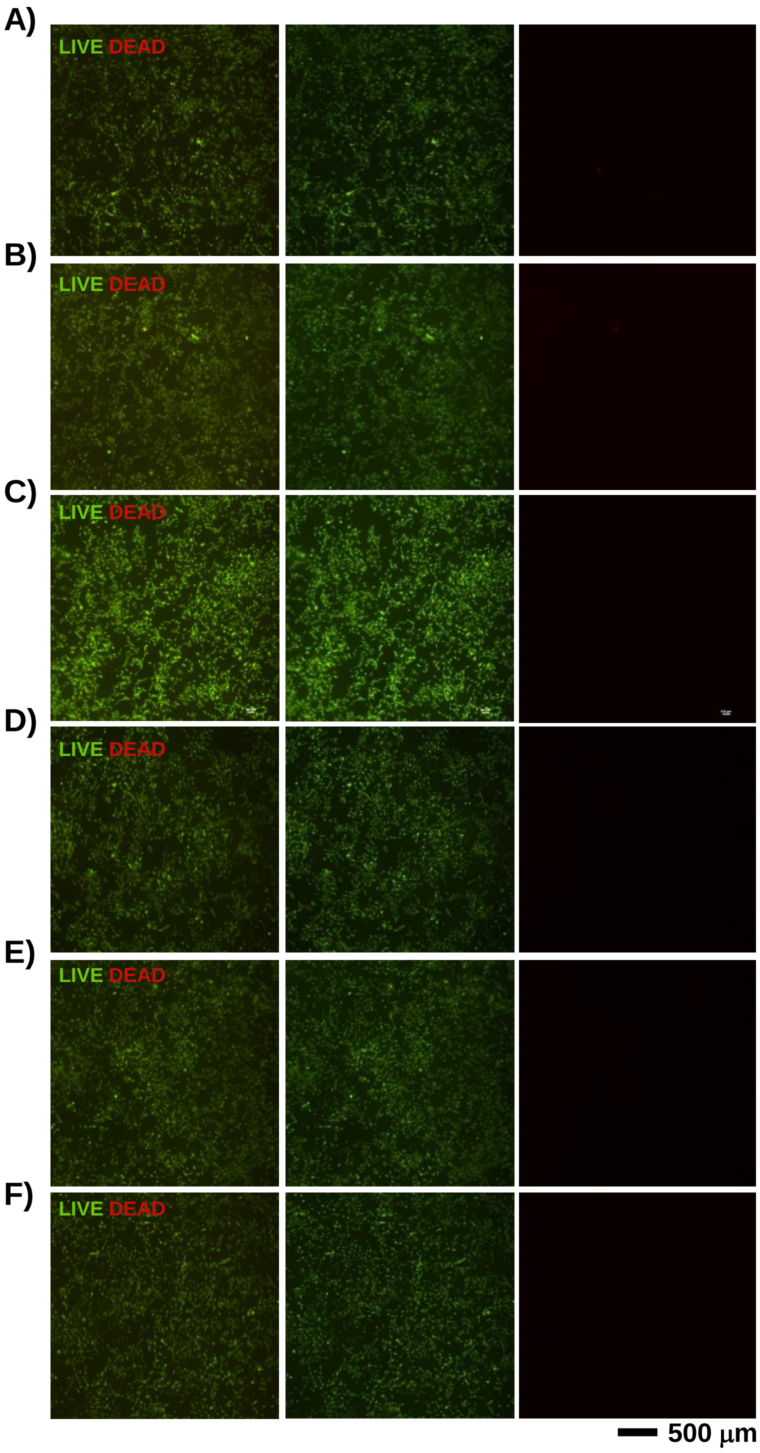

Figure S14. Representative live/dead fluorescence images of SMCs cells cultured with the hydrogel.

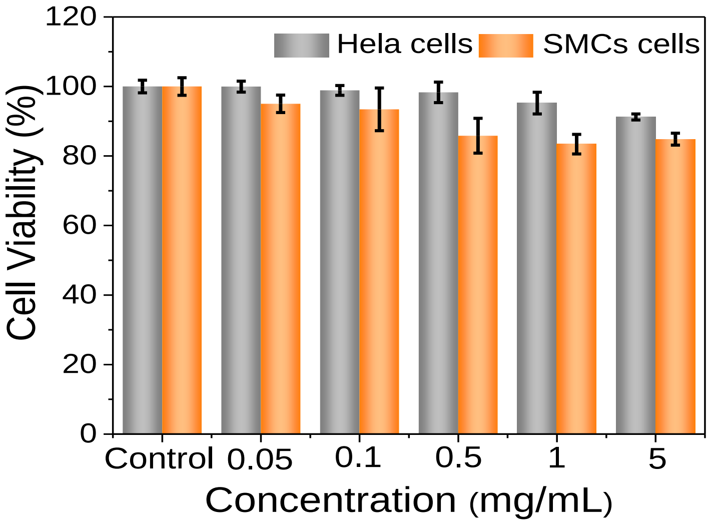

Figure S15: The cytotoxicity test of the hydrogel.

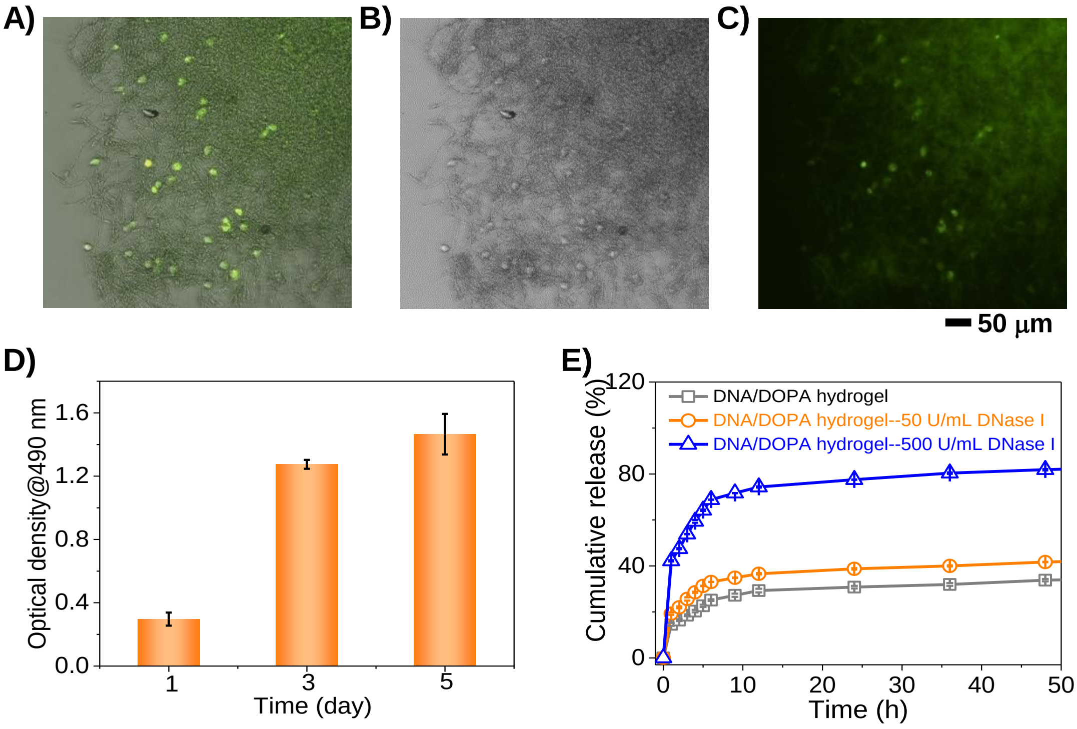

Figure S16: Cell adhesion and controlled drug release experiments of the hydrogel.

Table S1. The summary and comparison of our prepared hydrogel with the previously-reported swelling materials.

Substance	Modulus	Response time	Sensitivity upon solvents with a visible volume (shape) change	Ref.
p(DMAA-co-DMA) polymer hydrogel	100 KPa	5 min	The change of polarity: 3	[8]
poly(3-cyanomethyl-1-vinylimidazolium bis(trifuor omethanesulfonyl)imide)/carboxylic acid-substitute d pillar[5]arene composite membrane	--	A few seconds	The change of polarity: 4.8	[9]
silver NPs/polyacrylamide hydrogel	0.6-3 MPa	A few minutes	The change of polarity: 3.6	[10]
PNIPAm/PHEAm/cellulose nanocrystals hydrogel	2000 Pa	A few minutes	The change of polarity: 1.18	[11]
PNIPAAm/PNIPAAm- 2-(30,30-dimethyl-6-nitros piro [chromene-2,20-indoline]-10-yl) ethyl methacr ylate hydrogel	KPa level	A few minutes	The change of polarity: 5.9	[12]
hydrophobic poly(methyl acrylate) (PMA) gels	150 KPa	A few hours	The change of polarity: 3	[13]
PEG-DA hydrogel	50-400 KPa	A few hours	The change of polarity: 6.8	[14]
DEX-g-DOPA) and DNA	59 Pa	A few seconds	The change of polarity: 0.4	Our work

References

[1] R. Merindol, S. Loescher, A. Samanta, A. Walther, *Nat. Nanotechnol.* **2018**, *13*, 730.
[2] S. Mark, K. M. G. Taylor, D. Q. M. Craig, P. Karen, R. Hazel, *Pharm. Res.* **2007**, *24*, 1954.
[3] K. Kummer, D. V. Vyalikh, G. Gavrila, A. B. Preobrajenski, A. Kick, M. Bönsch, M. Mertig, S. L. Molodtsov, *J. Phys. Chem. B.* **2010**, *114*, 9645.
[4] I. L. Volkov, A. Smirnova, A. A. Makarova, Z. V. Reveguk, R. R. Ramazanov, D. Y. Usachov, V. K. Adamchuk, A. I. Kononov, *J. Phys. Chem.* **2017**, *121*, 2400.
[5] M. Shin, J. H. Ryu, J. P. Park, K. Kim, J. W. Yang, H. Lee, *Adv. Funct. Mater.* **2015**, *25*, 1270.
[6] M. M. Mady, W. A. Mohammed, N. M. El-Guendy, A. A. Elsayed, *Int. J. Phys. Sci.* **2011**, *6*, 7328.
[7] S. Kim, K. J. You, Y. Wang, H. Lee, J. W. Choi, *Adv. Mater.* **2018**, *30*, 1707594.
[8] Huang, Jiahe, Liao, Jiexin, Wang, Tao, Sun, Weixiang, Tong, Zhen, *Soft Matter.* **2018**, *14*, 2500.
[9] Q. Zhao, J. W. C. Dunlop, X. Qiu, F. Huang, Z. Zhang, J. Heyda, J. Dzubiella, M. Antonietti, J. Yuan, *Nat. Commun.* **2014**, *5*, 4293.
[10] H. Qin, T. Zhang, N. Li, H.-P. Cong, S.-H. Yu, *Nat. Commun.* **2019**, *10*, 2202.
[11] X. Wang, H. Huang, H. Liu, F. Rehfeldt, X. Wang, K. Zhang, *Macromol. Chem. Phys.* **2019**, *220*, 1800562.
[12] X. Zhang, X. Xu, L. Chen, C. Zhang, L. Liao, *Dyes Pigments.* **2020**, *174*, 108042.
[13] H. Guo, T. Nakajima, D. Hourdet, A. Marcellan, C. Creton, W. Hong, T. Kurokawa, J. P. Gong, *Adv. Mater.* **2019**, *31*, 1900702.
[14] B. M. Bailey, V. Hui, R. Fei, M. A. Grunlan, *J. Mater. Chem.* **2011**, *21*, 18776.
